# The SRC-family tyrosine kinase HCK shapes the landscape of SKAP2 interactome

**DOI:** 10.18632/oncotarget.24424

**Published:** 2018-02-06

**Authors:** Jean-François Bureau, Patricia Cassonnet, Laura Grange, Julien Dessapt, Louis Jones, Caroline Demeret, Anavaj Sakuntabhai, Yves Jacob

**Affiliations:** ^1^ Unité de Génétique Fonctionnelle des Maladies Infectieuses, Département Génome et Génétique, Institut Pasteur, Paris, France; ^2^ CNRS URA3012, Paris, France; ^3^ Unité de Génétique Moléculaire des Virus à ARN, Département Virologie, Institut Pasteur, Paris, France; ^4^ UMR3569, Centre National de la Recherche Scientifique, Paris, France; ^5^ Université Paris Diderot, Paris, France

**Keywords:** luciferase complementation assay, protein-protein interaction, SRC-kinase family, adaptor, FYB, Pathology

## Abstract

The SRC Kinase Adaptor Phosphoprotein 2 (SKAP2) is a broadly expressed adaptor associated with the control of actin-polymerization, cell migration, and oncogenesis. After activation of different receptors at the cell surface, this dimeric protein serves as a platform for assembling other adaptors such as FYB and some SRC family kinase members, although these mechanisms are still poorly understood. The goal of this study is to map the SKAP2 interactome and characterize which domains or binding motifs are involved in these interactions. This is a prerequisite to finely analyze how these pathways are integrated in the cell machinery and to study their role in cancer and other human diseases when this network of interactions is perturbed. In this work, the domain and the binding motif of fourteen proteins interacting with SKAP2 were precisely defined and a new interactor, FAM102A was discovered. Herein, a fine-tuning between the binding of SRC kinases and their activation was identified. This last process, which depends on SKAP2 dimerization, indirectly affects the binding of FYB protein. Analysis of conformational changes associated with activation/inhibition of SRC family members, presently limited to their effect on kinase activity, is extended to their interactive network, which paves the way for therapeutic development.

## INTRODUCTION

**Figure 1:**
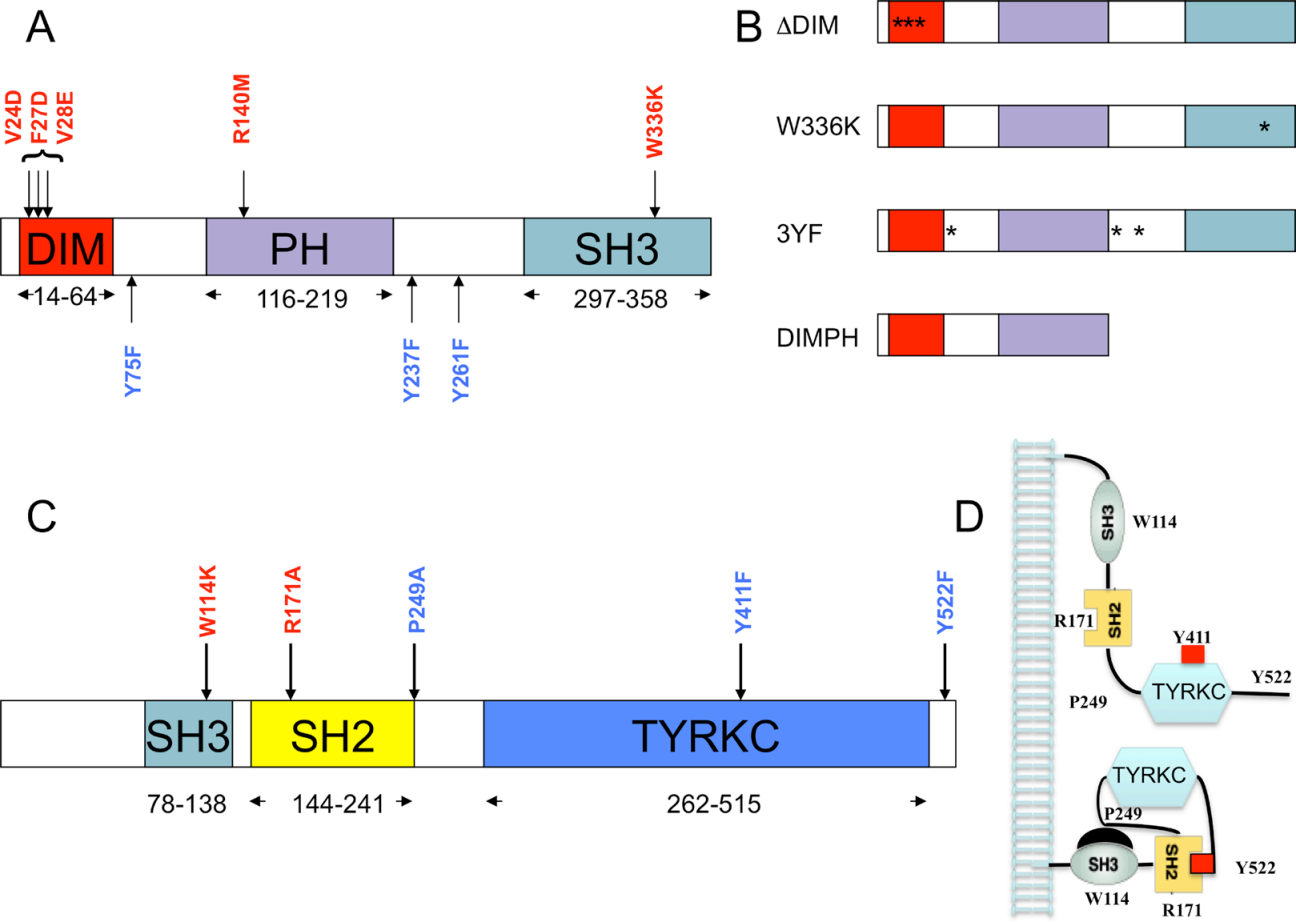
Structural domain organization of SKAP2 and HCK proteins For each protein, the delineation of domain boundaries is indicated. Wild type and replacement amino acid identities at the mutated position involved in domain or motif inactivation are indicated in red and blue respectively. (**A**) Schematic of SKAP2 organization with its three domains: dimerization domain (DIM), Plekstrin homology (PH) domain, and SRC homology 3 (SH3) domain. (**B**) Schematic of major SKAP2 mutants with their point mutations (star). (**C**) Schematic of HCK protein organization with its three domains: SRC homology 3 (SH3) domain, SRC homology 2 (SH2) domain and tyrosine kinase catalytic domain (TYRKC). (**D**) Schematic illustrating inactive (bottom) and active (top) forms of HCK.

The SRC Kinase Adaptor Phosphoprotein 2 (SKAP2), a broadly expressed protein conserved among gnathostomes and recruiting protein partners to specific subcellular domains, plays a central role in multiple physiological processes, including response to TGFβ [1], integrin signaling [2], control of actin-polymerization [3, 4], podosome stabilization, metastatic progression [5], and cell migration [6]. SKAP2 contains three domains: an N-terminal dimerization (DIM) domain, a Plekstrin homology (PH) domain, and a SRC homology 3 (SH3) domain (Figure 1A). Multiple sites of tyrosine phosphorylation are found across the entire protein [7], some of which match SRC homology 2 (SH2) binding motifs [8]. As shown by co-immunoprecitation, SKAP2 interacts with FYN, HCK, and LYN, members of the SRC kinase family [9, 10]. Murine Skap2 is a homodimer containing four-helix bundle dimerization (DIM) domains. These helix domains also interact with the PH domain of the same molecule, blocking access to its phosphatidylinositol [3–5] P3-binding pocket [11]. In *Skap2*^-/-^ mice, functions of dendritic cells and B cells, such as migration, are adversely affected [12–14]. In most hematopoietic cells, Skap2 interacts with the Riam-Fyb complex as Skap1, a paralog playing a non-redundant functional role [15]. This complex stabilizes the TCR at the immune synapses. SKAP2 directly interacts with FYB and with PTK2B proteins through its SH3 domain [16, 17]. Recently SKAP2 has been shown to promote podosome formation and to facilitate tumor-associated macrophage infiltration and metastatic progression [5]. High expression of SKAP2 is also associated with poor prognosis in non-small cell lung cancer [18]. This protein is also highly expressed in the CNS under the control of retinoic acid and during development [9]. In the lens of the eye, SKAP2 is a novel target of HSF4b, which associates with NCK2 adaptor but not with its NCK1 homolog [19].

The SRC kinase family is composed of the SrcA subfamily, with FGR, FYN, SRC, and YES, the SrcB subfamily with BLK, HCK, LCK, and LYN, and finally FRK [20]. A SRC-related kinase family lacking C-terminal regulatory tyrosine and N-terminal myristoylation sites and containing SRMS has been also described. SRC kinases phosphorylate tyrosine residues of proteins, and have a modular architecture composed of three domains, a SH3 domain, a SH2 domain and a tyrosine kinase catalytic domain (TYRKC) (Figure 1C). Their N-terminal region contains myristoylation and palmitoylation sites, which affect protein localization in different membranous subcellular compartments [21, 22]. Most SRC kinases are negatively regulated by intra-molecular interactions (Figure 1D) where the SH3 domain interacts with a proline-rich region located between the SH2 and the TYRKC domains, and the SH2 domain interacting with a C-terminal binding motif containing a phosphorylated tyrosine [23–25]. Dephosphorylation of this tyrosine or displacement of the SH3 domain induces conformational changes that partially activate the SRC kinases. The trans-phosphorylation of a tyrosine inside the TYRKC domain stabilizes this activation [26]. The SRC kinase HCK has the most complete structure, which excludes only the N-terminal 79 residues [27, 28]. At least seven groups of conformations have been detected for HCK protein, with the equilibrium between them modified depending on the binding of different signaling peptides [29]. Due to the role of SRC tyrosine kinases in oncogenesis, small molecule inhibitors of them have been extensively studied [30]. Several have become approved drugs for treatment of neoplastic disorders [25].

To better understand how adaptor proteins and SRC kinases interact with SKAP2, we carried out two complementary approaches: binary interaction screening by yeast two-hybrid and protein association screening by luciferase complementation assay in human cells. Yeast two-hybrid screening detected a previously unknown interactor of SKAP2, FAM102A, an early estrogen-induced adaptor which is also a component of the RANK signaling pathway essential for differentiation of osteoclasts [31, 32]. Luciferase complementation assay [33] coupled with site-directed mutagenesis confirmed this interaction, and also delineated domains or binding motifs for most of the 17 other candidate proteins interacting with SKAP2 selected by literature curation. Mutations in the HCK kinase known to affect its enzymatic activity also modified its binding to SKAP2, suggesting that these processes are coupled. Interaction of SRC family members with SKAP2 did not mostly involve the SH2 domain as previously suggested, but probably the SH3 domain, as shown here for HCK. Analysis of conformational changes with activation or inhibition of SRC family members was limited to their effect on kinase activity [34–36] and the present study extends it to their binding properties by using the luciferase complementation assay. This report opens a new path to analyze the effect of conformational changes on protein-protein interaction (PPI) network, with possible therapeutic implications [25, 30].

## RESULTS

### Assembling a set of SKAP2 interactors based on yeast two-hybrid screen and literature curation

Yeast two-hybrid screen identified FAM102A as a putative SKAP2 interactor. By combining this approach and literature curation, we assembled a set of 17 proteins as putative SKAP2 interactors. Six were adaptor proteins: FAM102A, FYB [10, 16], APBB1IP (also known as RIAM) [12], and NCK2 [19]; NCK1 and FAM102B because of their homology with NCK2 and FAM102A proteins, respectively. Ten putative interactors were members of the SRC tyrosine kinase family: FGR, FYN, SRC, and YES in the SrcA subfamily, BLK, HCK, LCK, and LYN in the SrcB subfamily, and finally FRK and one related kinase, SRMS. SRC kinases were selected as putative interactors because some of them have been described as SKAP2 interactors [9, 10, 16]. The last putative interactor is SKAP2 itself because its mouse ortholog is known to form a dimer [11]. We used luciferase complementation assay GPCA [33] to validate the findings of the yeast two-hybrid screen and of the literature curation.

This luciferase complementation protein-protein interaction (PPI) detection assay, done in HEK293T human cells, is based on complementation of split *Gaussia princeps* luciferase (GPCA). Each of the two hemi-luciferases is fused to SKAP2 and its putative interactor at either the N-terminal or C-terminal end. In the rest of the paper, we used the following nomenclature for hemi-luciferase fused proteins: N or C determines its localization, and 1 or 2 the hemi-luciferase chosen. Interaction is monitored by the luminescence induced by the reconstituted active *Gaussia princeps* enzyme. To minimize the possibility that the fusion position affects the stability of the SKAP2 dimer, we first tested the four possible configurations of the SKAP2-SKAP2 interacting pair (Supplementary Figure 1A). The results indicate that dimerization is not affected by the position of the *Gaussia* hemi-luciferase tag, as measured by normalized luminescence ration (NLR) values. The accuracy and the sensitivity of GPCA were evaluated against a set of ten random human proteins and a positive reference set of three known interacting partners, FYB, FYN, and LYN [9, 10, 16]. The signal of each of the three positive proteins interacting with SKAP2 is higher than those of each of the ten random proteins, as indicated by NLR values (Supplementary Figure 1B, left panel). The signal detected for FAM102B interaction with SKAP2 was always below that of the three positive controls but among the highest of the random controls (Supplementary Figure 1B, right panel). For that reason in the rest of the experiments, we decided to use FAM102B as a negative control associated with one or two random human proteins chosen among STAP1, SGTB, CRSP3, ZC3, and PCMT1. This approach defined a threshold for positive PPI in each experiment as the highest value of the NLR mean + SEM among negative controls. To test the effect of cell activation, HEK293T cells were also treated for 2 h 15 min with 5 µM A23187 molecule, a calcium ionophore, before PPI measurements.

### GPCA validation of SKAP2 interactors

**Figure 2:**
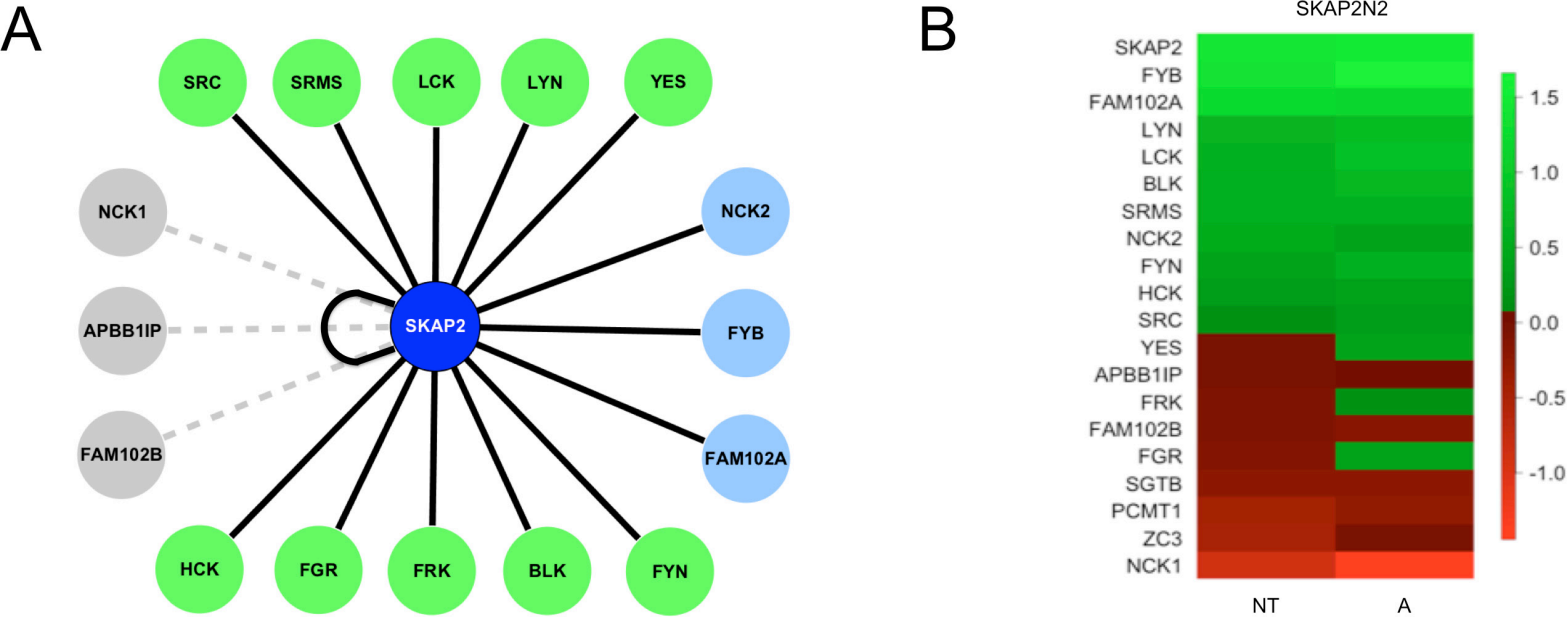
Literature curated interactions recovered with Gaussia princeps luciferase complementation assay (**A**) Cytoscape [46] schematic of SKAP2 protein-protein interactions (PPI) interactome network highlighting domains and their binding motifs. Node color indicates SRC family members (green), adaptors (blue) and non-interacting proteins (grey). PPIs that have been recovered are indicated with black edge, and lack of interaction with grey dotted edge. (**B**) Heatmap showing the protein-protein interactions of SKAP2N2 with putative partners detected by luciferase complementation assay. Scoring is based on logtransformation of normalized relative luminescence (NLR) intensity and the null value corresponds to the threshold. Interacting pairs are ranked high to low with strongest in green and lowest in red. Interactions are monitored on non-stimulated (NT) or A23187 calcium ionophore activated (A) cells.

A positive interaction was defined as repeatable NLRs above the threshold in at least one of the two localizations of *Gaussia princeps* fusion on SKAP2, with or without activation by A23187 calcium ionophore. Of the 17 putative interactors of SKAP2, 14 were validated (Figure 2A). Only APBB1IP, FAM102B, and NCK1 did not show interaction with SKAP2 in these conditions. A heat map summarizes the interaction of SKAP2N2 with the 17 putative SKAP2-interacting proteins (Figure 2B. Supplementary Table 1). Three proteins, FYB, SKAP2, and FAM102A, showed the strongest interactions with SKAP2. There was great variability of NLRs among members of SRC kinase family, with three of those proteins positive only after activation, YES, FRK and FGR. The interaction signals for the APBB1IP and NCK1 proteins were similar to those of the *a priori* non-interacting proteins FAM102B, PCMT1, SGTB, and ZC3. The interaction signal detected for some proteins increased with activation. Results were similar with SKAP2C2 (Supplementary Figure 1C, Supplementary Table 1). Again the interaction strengths for APBB1IP, and NCK1 proteins were similar to those of the *a priori* non-interacting proteins FAM102B, PCMT1, and SGTB. The interaction signal of FYB protein was similar regardless of the N-terminal or C-terminal localization of the SKAP2 fusion. For the remaining proteins tested, interaction with SKAP2 was greatly reduced when SKAP2 was fused C-terminal to the *Gaussia princeps* fragment 2 (SKAP2C2), with total loss of signal for FRK and SRC kinases. Based on this observation for SKAP2, N-terminal fusion was mainly used for further interaction experiments, and C-terminal fusion when mentioned was reserved to test few specific interactomic configurations. In contrast, for the different SRC kinase members, interactions were detected regardless the fusion position. We observed a strongest interaction for the SrcB subfamily members and the SRMS related kinase than for the SrcA subfamily members and FRK kinase (Figure 2B and Supplementary Figure 1C).

### Role of the dimerization domain of SKAP2

**Figure 3:**
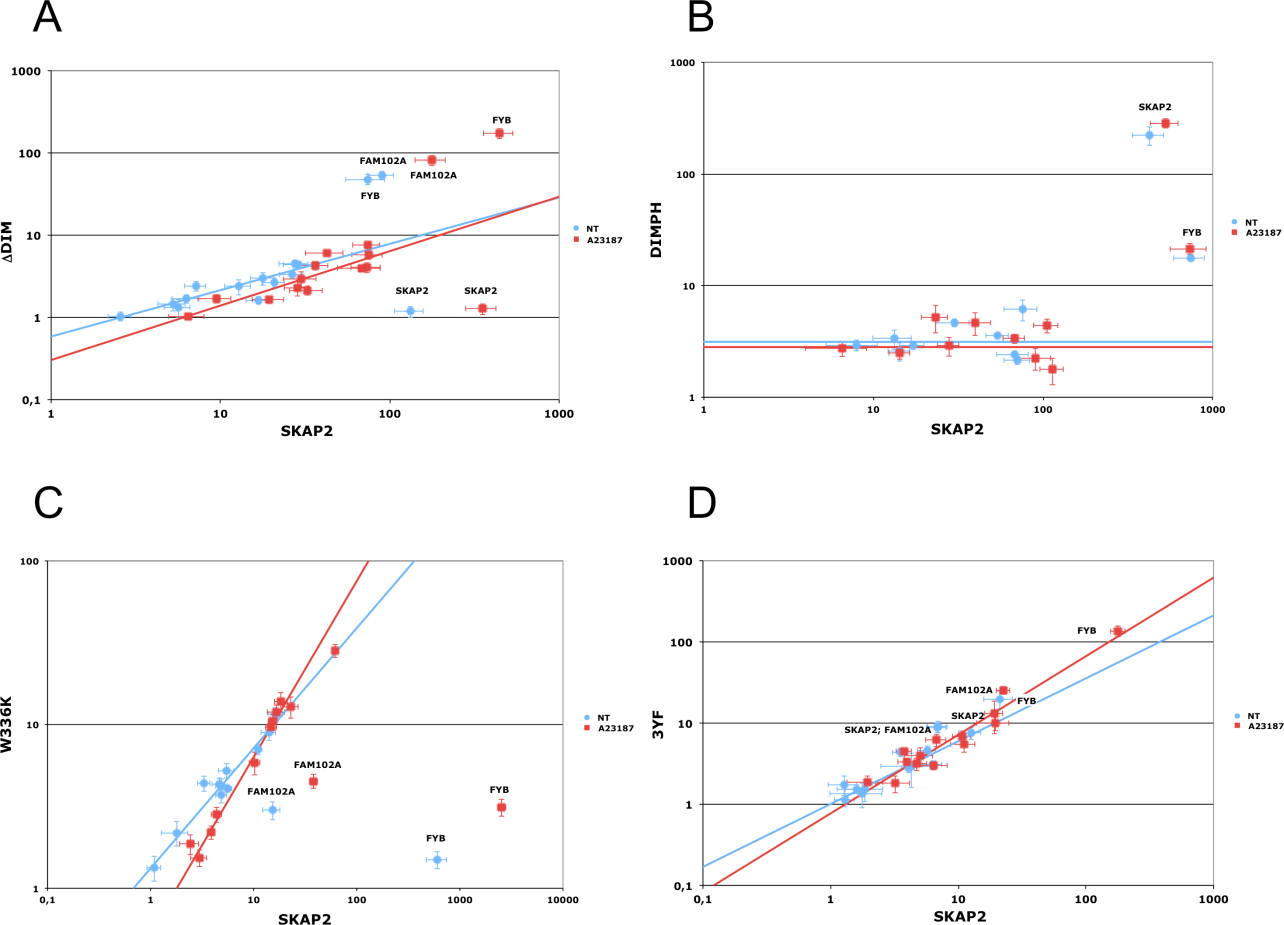
Edgetic effect of SKAP2 domain or binding motif inactivating mutations Differential interaction scatterplot of SKAP2 mutant (y-axis) versus SKAP2 wild-type (x-axis) using normalized luminescence ratios are displayed in (**A–D**). Interactions not affected by the mutation are aligned on the diagonal. PPI disruptive mutations are located in the lower right-hand quadrant in contrast to mutations stabilizing interaction that are located on the upper left-hand one. PPI affected by the mutation are annotated. Interactions are monitored on non-stimulated (blue circle) or A23187 calcium ionophore activated (red square) cells. Error bar: Standard Error to the Mean (SEM). A robust linear regression, which takes account of outliers, was performed on data using mmregress Stata module. (A) Scatterplot comparing the interactome of SKAP2N2 and its N2-fused ΔDIM mutant. Linear regression equations are respectively log10(ΔDIM) = 0.563 * log10(SKAP2) – 0.232 for samples without A23187 stimulation (blue line) and log10(ΔDIM) = 0.661 * log10(SKAP2) – 0.518 for samples with A23187 stimulation (red line). (B) Characterization of the DIMPH mutant. Scatterplots compare the interactome of SKAP2N1 and its N1-fused DIMPH mutant with N1-fused SRC family members. Threshold for data without (blue line) and with (red line) activation by A23187. (C) Scatterplot comparing the interactome of SKAP2C2 and its C2-fused W336K mutant. Linear regression equations are respectively log10(W336K) = 0.733 * log10(SKAP2) + 0.124 for samples without A23187 stimulation (blue line) and log10(W336K) = 1.072 * log10(SKAP2) – 0.269 for samples with A23187 stimulation (red line). (D) Scatterplot comparing the interactome of SKAP2C2 and its C2-fused 3YF mutant. Linear regression equations are respectively log10(3YF) = 0.775 * log10(SKAP2) – 0.001 for samples without A23187 stimulation (blue line) and log10(3YF) = 0.968 * log10(SKAP2) – 0.111 for samples with A23187 stimulation (red line).

In the mouse Skap2 ortholog the triplet mutations V24D, F27D, and V28E affects dimerization of Skap2 [11]. We introduced the corresponding triplet mutation into SKAP2 to generate the ∆DIM SKAP2 mutant (Figure 1A and 1B). Dimerization of ∆DIM SKAP2, as detected with GPCA, was specifically perturbed compared to SKAP2 wild type, independently of the activation by the calcium ionophore A23187 (Figure 3A, Supplementary Table 2). As figured in this differential interaction scatterplot, comparing the NLR values of proteins interacting with the ΔDIM SKAP2 mutant (y-axis) to that of proteins interacting with the SKAP2 wild-type protein (x-axis) with (red square) and without (blue circle) activation, the NLRs values of SKAP2 were located in the lower right-hand quadrant illustrating a strong decrease of SKAP2 dimerization signal with ΔDIM SKAP2. A robust linear regression used to detect outliers is in agreement with this result. Similar results were obtained with SKAP2C2 or C1-fused SRC kinase members (Supplementary Figure 2A and 2B).

### Binding of SKAP2 with FAM102A, and FYB or with SRC family members is affected differently by its dimerization

In addition to SKAP2, the NLR values of FAM102A and FYB were not aligned with SRC kinase members or the negative controls (Figure 3A), suggesting that SKAP2 dimerization differentially affects the binding of these two adaptors with SKAP2 as compared to the binding of SRC kinase members. The strength of the interaction with FAM102A and FYB seemed stronger than that with the SRC kinase members for the ΔDIM SKAP2 mutant compared to SKAP2 wild-type protein. C1-fused SRC kinase members (Supplementary Figure 2A and Supplementary Table 3) or SKAP2C2 (Supplementary Figure 2B and Supplementary Table 4) gave similar results, ruling out an effect of position of the hemi-luciferase on the results.

We decided to construct a SKAP2 mutant that still dimerizes with similar efficiency than SKAP2 wild-type but has lost its capacity to bind to FYB, FAM102A, and SRC family members. We constructed a mutant with the first 222 amino acids of SKAP2, the DIMPH segment, containing both the DIM and the PH domains and removing not only the SH3 domain but also two of the three tyrosines of putative SH2 binding motifs (Figure 1B). We first compared interaction with the DIMPH deletion mutant to interaction with SKAP2 wild-type protein (Figure 3B and Supplementary Table 5). The DIMPH deletion mutant interacted with SKAP2 similarly to SKAP2 wild-type protein (or slightly weaker), independently of the activation by A23187 molecule (see also Supplementary Table 5). In contrast, the strength of the interaction with FYB strongly decreased, and no interaction or very weak interaction occurred between the DIMPH deletion mutant and N1-fused SRC family members, although some weak binding was detectable with C1-fused SRC family members (Supplementary Figure 3 and Supplementary Table 5).

We tested whether the dimerization of SKAP2 affects the binding of FYB and FAM102A as compared to that of the SRC family members. First, a competition experiment (Supplementary Figure 4A) showed that the strength of the interaction of SKAP2 for FYB and possibly FAM102A was slightly stronger than that of the other proteins tested (SKAP2, SRC family members, negative controls) in presence of the DIMPH deletion mutant compared to its absence (Supplementary Figure 4B, Supplementary Table 6). This effect is weaker than that of the ΔDIM SKAP2 mutant (Supplementary Figure 4C, Supplementary Table 6). These first results are in agreement with a differential binding due to SKAP2 dimerization even if the effect is weak.

Second, to detect ternary protein complexes, we used HaloTag technology [37] (Promega Wisconsin). In the strategy, one protein is fused to HaloTag as a standardized “hook”, used to capture on HaloLink™ resin in which a HaloTag ligand has been covalently bound the protein pair generating the luciferase signal. The goal is to compare the binding of FYB to either SKAP2 homo-dimer or SKAP2-DIMPH mutant hetero-dimer, where only the SKAP2 chain is able to bind both SRC kinase family members and FYB (Supplementary Figure 5). Complexes with FYB and SKAP2 homo-dimers had lower luminescence ratio than those with FYB and SKAP2-DIMPH hetero-dimers (Table 1) in agreement with differential interaction scatterplot data (Figure 3A). This result has been repeatedly found independently of the protein fused to HaloTag, either SKAP2 or FYB. Although, the luminescence of the complex between HaloTag-SKAP2, SKAP2, and FYB was repeatedly lower than that of the complex between HaloTag-DIMPH, SKAP2, and FYB. In contrast in Figure 3B (Supplementary Table 5), the luminescence of DIMPH-SKAP2 hetero-dimer was similar or slightly lower that of SKAP2 homo-dimer. These opposite results do not support that the difference of luminescence detected during the ternary protein complex experiment is due to a difference of efficiency to form dimer between DIMPH and SKAP2 wild-type. In summary, these experiments strongly support that FYB interacts with a higher efficiency to the SKAP2-DIMPH hetero-dimer than to the SKAP2 homo-dimer. As described below, the W336K SKAP2 mutant has no SH3 function and no or poor interaction with FYB and FAM102A proteins. Complexes with FYB and SKAP2-W336K SKAP2 hetero-dimers, in which only SKAP2 chain is able to bind FYB but both chains bind SRC kinase members, had similar luminescence ratio than the complexes with FYB and SKAP2 homo-dimers (Table 1), suggesting that the increase of the luminescence ratio for complexes with FYB and SKAP2-DIMPH hetero-dimer is not due to the binding of FYB but to other proteins such as the SRC family members.

**Table 1:** Detection of ternary protein complexes

Complex name	Experiment 1 Luminescence ratio^*,°^	Experiment 2 Luminescence ratio^*,°^	Experiment 3 Luminescence ratio^*,°^
HTSKAP2_SKAP2_FYB	1.33	8.63	1.05
HTSKAP2_DIMPH_FYB	6.38 (480%)	26.78 (310%)	11.68 (1112%)
HTSKAP2_SKAP2W336K_FYB	ND	ND	4.05
HTDIMPH_SKAP2_FYB	14.45	15.69	ND
HTFYB_SKAP2_SKAP2	49.08	134.99	51.78
HTFYB_DIMPH_SKAP2	124.78 (254%)	308.45 (229%)	160.29 (310%)
HTFYB_SKAP2W336K_SKAP2	ND	63.79	11.08
Control	ND	0.89	ND

^*^ND: not done

°Percent of increased compared to the complex in which SKAP2 replace DIMPH

### Proteins interacting with the SH3 domain of SKAP2

A W336K point mutation in the double tryptophan of the SH3 domain is known to impair its binding to proline-rich proteins such as FYB [38]. This mutation was generated on both SKAP2N2 and SKAP2C2 fusion protein. Due to a noisy signal of the W336K SKAP2N2 mutant, interaction strength was measured only for SKAP2C2. The sensitivity of the assay was also increased by using C1-fused SRC family members. Interaction of FAM102A and FYB adaptors with SKAP2 was specifically reduced in the W336K SKAP2 mutant compared to SKAP2, independently of the activation by the A23187 calcium ionophore (Figure 3C, Supplementary Table 7). These results confirmed that the binding of SKAP2 to FYB occurred mainly through the SH3 domain of SKAP2, and lends support to a similar role of this SKAP2 SH3 domain for binding to FAM102A. We excluded a position effect by comparing interaction strength of N2-fused W336K SKAP2 mutant with FYB, SKAP2, FAM102A proteins and HCK mutants, all chosen for their strong interaction with SKAP2, to those of SKAP2N2 (Supplementary Figure 6A). A P149L mutation of the FAM102A adaptor protein, which is located inside two putative SH3 binding motifs (PxxP149LxR and PxxP149LxxP) [8], decreased interaction signal between FAM102A and SKAP2 adaptors, independently of the activation by A23187 molecule (NT: *z* = 6.07 *P* < 0.001; A: *z* = 5.25 *P* < 0.001; Supplementary Figure 6B, see also Supplementary Table 8). A 21-aa deletion of the second proline-rich region of FYB (amino acid 354 to 375) drastically reduced interaction signal between FYB and SKAP2 (NT: *z* = 6.50 *P* < 0.001; A: *z* = 11.93 *P* < 0.001) as previously reported [39]. The P370A mutation of FYB, which is located inside a putative SH3 binding motif in the same region of the protein, only partially diminished interaction (NT: *z* = 5.21 *P* < 0.001; A: *z* = 10.42 *P* < 0.001).

### Proteins interacting with the three SH2 binding motifs of SKAP2

Phosphorylation of the three tyrosines Y75, Y237, and Y261 has been reported to create SH2 binding motifs for SKAP2 [7, 8]. Co-immunoprecipitation experiments have affirmed that the Y261 phosphorylation is necessary for interaction between some SRC kinase members and SKAP2 [1]. We generated three SKAP2 mutants, each bearing one of the Y261F, Y237F, and Y75F mutations (Figure 1A). The binding of SRC family members to SKAP2 was not modified for any of these mutants (data not shown). In contrast, interaction of the NCK2 adaptor was decreased in the Y75F SKAP2 mutant compared to SKAP2 [19] (Supplementary Figure 7A, see also Supplementary Table 9). A role in protein-protein interaction of this SH2 binding motif of SKAP2 was confirmed with the NCK2-R311A mutation, in which the SH2 domain of NCK2 is inactivated. The R311A NCK2 mutant interacted weaker with SKAP2 than NCK2 wild type (NT: *z* = 1.98 *P* < 0.05; A: *z* = 2.86 *P* < 0.01; Supplementary Figure 6B). We also studied the triplet 3YF SKAP2 mutant bearing the three mutations, Y261F, Y238F, and Y75F. The binding strength of the different SRC family members to SKAP2 was not affected by this triplet mutation (Figure 3D, Supplementary Table 10).

### How SRC family members interact with SKAP2

**Figure 4:**
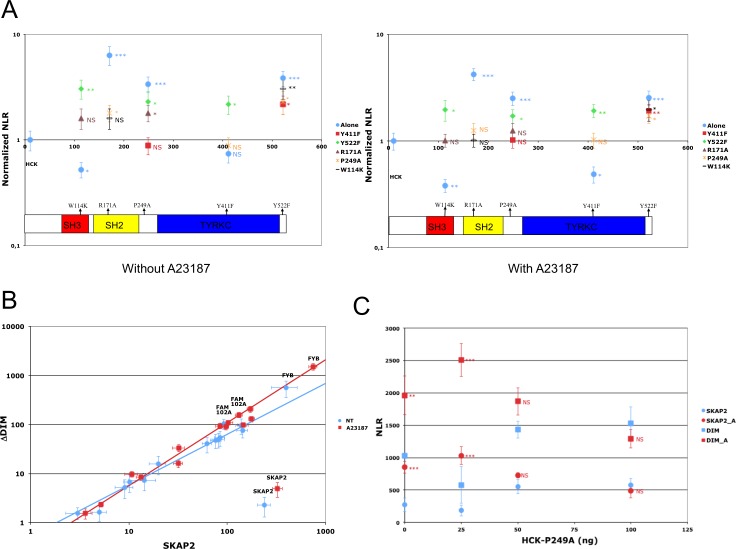
Impact of HCK mutations on SKAP2 interactions (**A**) The PPI-mutation plot summarizes results. Y-axis represents NLR of each N1-fused HCK mutant and X-axis, the position of the mutation without (left) and with (right) A23187 molecules. NLR are normalized according to that of HCK-N1. (**B**) Role of HCK mutants on the difference of binding due to SKAP2 dimerization. Scatterplot compares ΔDIM SKAP2 mutant (y-axis) and SKAP2 (x-axis) using HCK mutants instead of SRC family members. See legend of Figure 3 for details on scatterplot. Previous annotated PPI are reported on this graph. Linear regression equations are respectively log10(ΔDIM) = 1.035 * log10(SKAP2) – 0.273 for samples without A23187 stimulation (blue line) and log10(ΔDIM) = 1.282 * log10(SKAP2) – 0.529 for samples with A23187 stimulation (red line). (**C**) Role of HCK titration on the interaction between FYB and SKAP2. The NLR of FYB with SKAP2 and its ΔDIM mutant is shown according to different quantities (ng) of P249A HCK mutant. NLR of activated and non-activated samples were compared using a *z*-test. Circle: NLR of SKAP2; square: NLR of ΔDIM mutant with (red) and without (blue) A23187 activation. Error bar: SEM. *P* < 0.05 (^*^); *P* < 0.01 (^**^); *P* < 0.001 (^***^); Non-Significant (NS).

Our data strongly suggest that the interactions between SKAP2 and the different SRC-kinase family members were not affected by tyrosine to phenylalanine mutations known to destroy putative SH2 binding motifs. We next studied different mutations in HCK protein, a SRC family member which has a well-documented 3D structure [27, 28] (Figure 1C). It was shown that an inactive form of HCK protein is stabilized by an intra-molecular interaction between Trp114 of the SH3 domain and Pro249, or by an intra-molecular interaction between Arg171 in the SH2 domain and the phosphorylated Tyr522. Furthermore, phosphorylation of Tyr411 was described to stabilize HCK conformation and its kinase activation [26, 40] (Figure 1C and 1D). We tested whether mutations of these five amino acids alone or in combination will affect the binding to SKAP2. Results are displayed on a PPI-mutation plot, which shows the wild-type HCK-normalized NLRs of the different HCK mutants depending on mutation position. Interestingly, most HCK mutants had stronger interaction with SKAP2 than wild type HCK (Figure 4A, Supplementary Table 11). Only mutations W114K and Y411F decreased the interaction signal between HCK and SKAP2. The SH3 domain of HCK plays a role in the binding to SKAP2, since the signal of P249A HCK mutant with a mutation inside the internal SH3 binding site was higher than that of W114K HCK mutant with a mutation inactivating its SH3 domain (NT: *z* = 11.49 *P* < 0.001; A: *z* = 11.24 *P* < 0.001; Figure 4A left-hand image and Supplementary Table 11). The increase in interaction strength with SKAP2 after SH3 domain displacement depends at least partially on the activation of HCK and its stabilization by phosphorylation of its Trp411 since the signal of P249A-Y411F HCK double mutant was lower than that of P249A HCK mutant (NT: *z* = 9.54 *P* < 0.001; A: *z* = 7.02 *P* < 0.001) and higher than that of Y411F HCK mutant (NT: *z* = 1.20 ns; A: *z* = 4.67 *P* < 0.001). Y522F and R171A HCK mutations affect the conformation of HCK protein and argue against a role of the SH2 domain for binding to SKAP2 since the signal of R171A HCK mutant with a mutation inactivating the SH2 domain was at least similar to that of Y522F HCK mutant with a mutation inactivating its internal SH2 binding site (NT: *z* = 2.79 P < 0.01; A: *z* = 6.22 *P* < 0.001) and both were greater than that of HCK wild type. Modification of the HCK conformation induced by each of the three mutations P249A, R171A, and Y522F changed how HCK interacts with SKAP2 for the following reasons. Firstly, the W114K R171A HCK double mutant, which has its two binding domains inactivated, bound SKAP2 similarly to HCK wild type. Secondly, W114K Y522F HCK double mutant interacts with SKAP2 similarly to Y522F HCK mutant, suggesting that its SH3 domain plays a minor role for the binding to SKAP2 after activation of HCK. These results supported appearance of new interacting sites between HCK and SKAP2 after the modifications of HCK conformation, one of which might be in its SH2 domain. Similar results were obtained by studying interactions between HCK mutants and SKAP2 fused with hemi-luciferase at its C-terminal end (Supplementary Figure 7B and Supplementary Table 12), excluding a position effect. The great differences in strength of binding to SKAP2 of SRC family members was not modified by mutating its three tyrosines, in opposition to previous results [1]. Most HCK mutations increased the binding to SKAP2, and support a direct role of the SH3 domain for interaction with SKAP2, at least in the initial step of the binding, without excluding a role for the SH2 domain in more active conformations. Most importantly, HCK mutations increasing the binding to SKAP2 are known to increase its kinase activity [26–28, 40] supporting that both functions are linked.

### HCK binding modifies the interaction strength between FYB and SKAP2

Dimerization status, which affects differently interactions between FYB or FAM102A and SKAP2 from those between the SRC family members and SKAP2 (Figure 3A), strongly decreased when HCK mutants replaced SRC family members (Figure 4B, Supplementary Table 12). These results suggest that the binding of SRC kinases to SKAP2 modifies the strength of binding for FYB and FAM102A. A direct proof for FYB and HCK is shown in Figure 4C. Both binding of HCK protein to SKAP2 and the calcium influx affected interaction strength between FYB and SKAP2. Without activation, NLR of FYB showed a U shaped curve suggesting that the binding of HCK protein to SKAP2 affects that of FYB. After activation, NLR showed a bell shaped curve suggesting a significant role of the activation status in binding. This combined effect of calcium and P228A HCK mutant on the interaction between FYB and SKAP2 occurred both with wild type SKAP2 and ΔDIM SKAP2 mutant.

We defined the domains or binding motifs of SKAP2 that interact with fourteen proteins. Dimerization of SKAP2 fine-tunes binding to SRC kinases and their activation that secondarily affects the binding of FYB protein.

## DISCUSSION

We developed a luciferase complementation assay to analyze interaction between SKAP2 and 17 putative protein partners. The normalized luminescence ratio depends not only on the strength of the interaction but also on the localization of each fragment of the *Gaussia princeps* luciferase. To minimize position effects, we analyzed the interaction of SKAP2 fused to *Gaussia princeps* fragment 2 at both the N and C terminus. Protein functions might be compromised by the position of the fusion protein, such as the plasma membrane localization of SRC family members by the fusion at their N-terminal end (Supplementary Figure 3). Fusion at the C-terminal end of SRC family members possibly affects their activation by interfering with phosphorylation of Trp522. To account for such effects, we studied SRC family members fused to *Gaussia princeps* fragment 1 at both ends. We used activation by A23187 molecule, a calcium ionophore, to test its effect on interaction strength. This effect was limited except for the interaction between FYB and SKAP2 with the P228A HCK mutant present. By examining mutants with point mutations, alone or in combination, we were able to precisely define the domain and its binding motifs necessary for these protein interactions (Supplementary Figure 8).

We confirmed the well-known interaction between the second proline-rich region of FYB adaptor and the SH3 domain of SKAP2 [10, 16, 39]. We also confirmed the interaction between the SH2 domain of NCK2 adaptor and the phosphorylated Tyr75 binding motif of SKAP2 [19]. We extrapolated from mouse Skap2 to the human SKAP2 that it can dimerize through its N-terminal region [11] and showed that the FAM102A adaptor interacts with the SH3 domain of SKAP2 by a binding motif containing Pro149. This result is in agreement with a previous report [41] showing that FAM102A protein interacts with SKAP1 protein, a paralog of SKAP2 mainly expressed in hematopoietic cells. We re-evaluated how SKAP2 interacts with SRC family members. We determined that the role of the different SH2 binding motifs of this protein has been over-estimated and that the SH3 domain of HCK and probably those of other SRC family members play an important role. Mutation of the three tyrosines, Y75F, Y237F, and Y261F, which inactivated putative binding motifs [7, 8], did not affect the great variability of interaction strength amongst SRC family members (Figure 3D and Supplementary Table 10). The results with HCK mutants strongly argue that the different conformations associated with kinase activation are responsible for the binding differences between SRC family members, with HCK activation directly affecting binding (Figure 4A and Supplementary Table 12). The stronger interaction strength for HCK mutants than for HCK protein could be explained by two non-exclusive events: increase in the number of HCK molecules able to interact with SKAP2 or increase in the binding capacity of each HCK molecule for SKAP2.

With the ΔDIM SKAP2 mutant, which does not dimerize, we detected a more subtle effect: dimerization increased the interaction strength of SRC family members compared to that of FYB and FAM102A proteins, independently of the orientation of the fused hemi-luciferase on SKAP2. Competition experiments with the DIMPH deletion mutant supports this hypothesis and also excludes a structural role for dimerization in the interaction. Results with HCK mutants argue that HCK activation affects its binding properties to SKAP2 (Figure 3A versus Figure 4B) and that Tyr411 phosphorylation is necessary for the interaction (Figure 4A).

**Figure 5:**
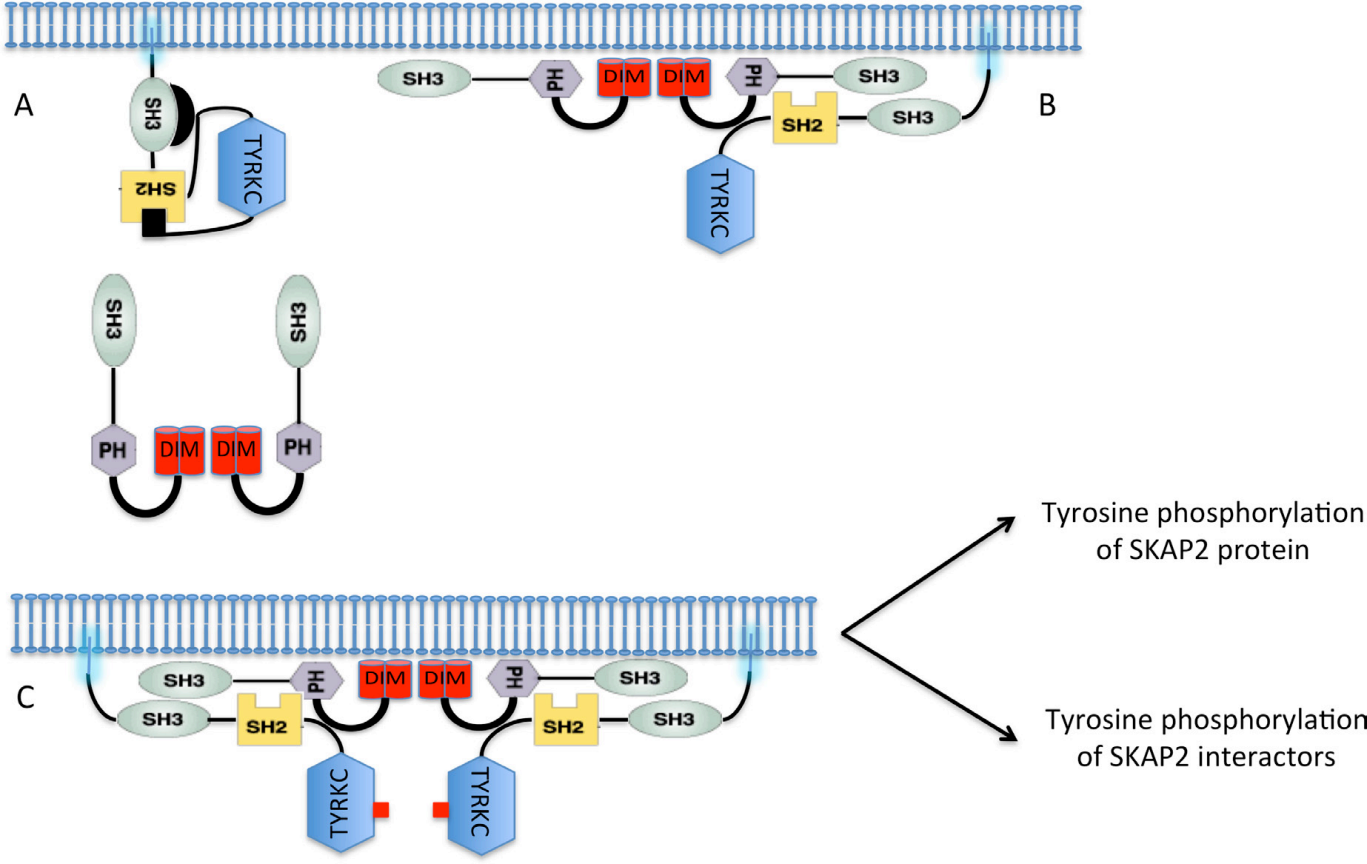
A model coupling binding of the SRC family members to SKAP2 to their activation (**A**) At the steady step, SRC kinase members are located to membrane and SKAP2 is mainly located to the cytoplasm. (**B**) At the beginning of activation, SKAP2 is now localized at the membrane after dissociation of chemical bond between its DIM and PH domains and binds one SRC kinase member. (**C**) SRC kinase members are fully activated when 2 molecules bind together SKAP2 dimer inducing trans-phosphorylation of a tyrosine stabilizing the activation of the kinase domain. Black rectangle: phosphorylation on tyrosine residue located at the C-terminal part of the SRC kinase member and inhibiting the kinase activity. Red rectangle: Activation of the TYRKC domain.

We propose the following model (Figure 5) for SKAP2 interaction. When two SRC family members bind to the same SKAP2 dimer, they activate in trans via phosphorylation of Tyr411 [26, 40]. This activation induces conformational changes that increase the binding of the SRC kinase or tyrosine phosphorylation of SKAP2, which increases the number of SH2 binding motifs. A similar model coupling activation of Src kinase with its binding to the integrin β cytoplamic domain has been already proposed [42]. This model has several notable consequences: 1) over-expression of SKAP2 induces a decrease of activity for trans-activated enzymes not because it inactivates these enzymes but because of titration; 2) Differences of binding among SRC kinases for SKAP2 might be explained not only by differences of binding capacity but also by differences of efficiency for conformational changes. This effect of SRC family members also modified the strength of FYB binding to SKAP2 even if the molecular events are unclear. Detection of ternary protein complexes definitively excluded the proposition that the strength of binding for FYB protein to SKAP2 depends on its SH3 domain and suggested a role of SRC family members. We definitely confirmed this role by studying the titration effect of P249A HCK mutant on the binding of FYB to SKAP2 and its ∆DIM mutant (Figure 4C).

From COSMIC cancer database [43], we extracted seven non-synonymous mutations detected repeatedly in *SKAP2* gene (Table 2). The *SKAP2*^E235*^ allele codes for a protein similar to the DIMPH deletion mutant but with twelve additional amino acids. In the heterozygous state, the DIMPH deletion mutant has a dramatic effect supporting a functional role of the E235* mutation. Also, a R140M mutation inactivates the PH domain [11], so the R140W mutation might have a similar effect. We evaluated the effect of two other mutations, V240I and I345V, but without any success. This negative result for the V240I SKAP2 mutation excludes the only potential SH3 binding motif for SRC family members predicted by ELM [8], and instead argues for a non-canonical SH3 binding motif mediating the interaction.

**Table 2:** Non-synonymous mutations detected repeatedly in *SKAP2* gene

Mutation^*^	Count^*^	putative mechanism
L118Q	2	
R126C	2	
R140W	2	R140M mutaion inactivates PH domain
E235^*^	3	Similar effects of DIMPH mutant
V240I	2	
I345V	2	

^*^from COSMIC cancer database

The luciferase complementation assay [33], developed for its good performance in the high-throughput setting, also enables fine exploration of complex PPIs. This assay combined with site-directed mutagenesis allowed us to validate protein-protein interaction, to precisely resolve the mechanisms of binding and to finely analyze protein conformations affecting the interactions. We evaluated the role of the different sub-domains of SRC family members on the binding to SKAP2 and we propose a model in which activation and conformational changes play a major role in controlling these interactions [44].

## MATERIALS AND METHODS

### Plasmids, mutagenesis, and BP cloning

The ORF of *SKAP2* (NM003930), *FYB* (NM001243093), *SRC* (NM005417), *APBB1IP* (NM019043) followed by a stop codon and flanked by two gateway sites have been synthesized and cloned in PUC57 plasmid (GenScript, Hong Kong). These four clones were individually transferred by Gateway recombinational cloning into pDONR207 vector. The ORF of *SKAP2* without stop codon and flanked by two gateway sites was also amplified by PCR, cloned into pCR-II TOPO^®^ vector using TOPO TA cloning kit (Thermo Fisher Scientific, Waltham) and transferred into pDONR207 vector. A similar procedure was developed for the *DIMPH* mutant, which contains the 5′ part of *SKAP2* ORF from the first to 666 bp and codes for the dimerization and the pleckstrin homology domains. Mutagenesis of *FAM102A*, *FYB*, *NCK2*, *SKAP2* and *HCK* ORFs were performed using QuickChange Lightning Site-Directed Mutagenesis kit (Agilent Technologies, Les Ulis) according to the manufacturer’s protocol. Supplementary Table 13 shows the primers used for all these purposes. After each mutagenesis and PCR amplification, the insert was completely sequenced using BigDye Terminator v3.1 Cycle Sequencing Kit (Thermo Fisher Scientific, Waltham) and 3130xl Genetic Analyzer (Applied Biosystems, Foster City).

### The yeast two-hybrid screening

We screened about 2 × 10^7^ yeast cells transfected with Human ORFeome v3.1 from which 4 10^6^ were diploids (17.7%) with SKAP2 pNY2H-GBK at 10 mM 3-aminotriazole (3-AT). One hundred and ninety two clones were randomly amplified and their insert sequenced. One hundred and forty six sequences out of 149 with a significant homology match FAM102A sequence. The 3 others single clones were considered as false positive.

### LR cloning and luciferase complementation assay

The four ORFs synthesized at GenScript and the thirteen ORFs from Human ORFeome 2011 were transferred by Gateway recombinational cloning into pSPICA-N1 vector. An ORF of SKAP2 without stop codon was also similarly transferred into pSPICA-C1 vector as the nine ORFs of the SRC kinase family from the Human ORFeome 2011. The ORFs of *SKAP2* with and without stop codon were also transferred into pSPICA-N2 and pSPICA-C2, respectively. The pSPICA vectors are mammalian expressing vectors designed for luciferase complementation assay. They expressed *Gaussia princeps* Luciferase fragment 1 (amino acid residues 18 to 109) or 2 (amino acid residues from 110 to 185) at the N or C-terminal part of the fusion protein.

The luciferase complementation assay was performed according to [33] with minor modifications. Briefly the first day, 30 000 HEK293T cells are cultured in 100 μl of DMEM supplemented with 10% Fetal Bovine Serum and antibiotics per well of microplate 96 wells (Greiner, Kremsmünster). One day later, cells were transfected with 100 ng GPCA plasmid pair using polyethylenimine (PEI) method. The third day, confluent HEK293T cells were stimulated if necessary by 5 µM A23187 during 2 h 15 min. Culture medium was discarded, cells were washed once with 150 μl of Phosphate Buffer Saline without calcium and magnesium (PBS) and incubated for 25 min in 40 μl of lysis buffer. Luminescence monitoring was performed after addition of native Coelenterazine on a Centro XS^3^ LB 960 microplate luminometer (Berthold Technologies, Thoiry). Transfections were performed at least in triplicate. Protein-protein interactions were monitored by measuring interaction-mediated normalized luminescence ratio (NLR). Approximations for mean and variance of a ratio were derived from Taylor expansion. For competition experiments, the ORF of *DIMPH* mutant was transferred by Gateway recombinational cloning into pCNeo vector. Transfection by PEI method was performed with 50 μg of each of GPCA vectors and 100 μg of the pCNeo vector containing either the ORF of *DIMPH* mutant or empty. Similar procedures were performed for titration experiment except that P249A HCK pCNeo was mixed with empty pCNeo. We used four quantities of P249A HCK pCNeo vector, 0 ng, 25 ng, 50 ng, 100 ng, completed to 100 ng using empty pCNeo vector.

### Detection of ternary protein complexes using a HaloTag vector

A gateway HaloTag vector (Promega, Madison) has been constructed by inserting the gateway cassette B at the blunted PvuI restriction site of pHTN HaloTag CMV-neo vector (Promega, Madison). The first day, 500 000 HEK293T cells were cultured in 2 ml DMEM supplemented by 10% FBS and antibiotics per well of plate 6 wells. The second day, cells were transfected by PEI method with 1 μg each of the HaloTag vector and the two GPCA vectors in 500 μl DMEM. For each sample, we used as control cells transfected with 1 μg pCNeo vector expressing the same protein than the HaloTag vector and the same two GPCA vectors. The third day, culture medium was discarded, cells were washed once with 2 ml Phosphate Buffer Saline without calcium and magnesium (PBS), incubated for 5 to 10 min in 900 μl of lysis buffer, and scraped. The medium is transferred to an Eppendorf tube and centrifuged 5 min at 5 K rpm to eliminate cellular debris. Forty μl of the supernatant was transferred into a 96 well microplate (Greiner, Kremsmünster). The rest of the supernatant was transferred to a new Eppendorf tube containing 40 μl HaloLink resin (Promega, Madison), and mixed during one hour on a wheel at maximum speed. Magnetic beads were purified on a DynaMag™-2 magnet (Thermo Fisher Scientific, Waltham), and submitted to 6 cycles of wash with 900ml PBS containing 0.1% Tween followed by magnet purification. The pellet was diluted in 40 μl lysis buffer and transferred into a 96 wells microplate (Greiner, Kremsmünster). Luminescence monitoring was performed as described above. Ternary protein complexes were monitored by the ratio of the luminescence from cells transfected with the HaloTag vector to that from cells transfected with the pCNeo vector.

### Graphic presentation and statistics

Approximations of the mean and the variance of a ratio were performed using Taylor expansions with a null covariance between the numerator and the denominator. Two-sample *z*-test is used to compare two of these ratios. Log-transformed NLRs of different proteins from the same experiment were compared using a histogram. Specific effects of mutations have been extensively studied using scatterplots: log-transformed NLRs of proteins interacting with SKAP2 mutant on the y-axis and those interacting with SKAP2 on the x-axis. NLRs of proteins not affected by the mutation were aligned in contrast to those interacting preferentially with SKAP2, which are located in the lower right-hand quadrant and those interacting preferentially with SKAP2 mutant, which are located in the upper left-hand quadrant. A robust linear regression using the rreg function of Stata/IC 10.1, a reweighted least squares algorithm based on M-estimators and Cook distances, was performed to define weight associated with each sample. Undefined or lower weights were used to confirm outliers by an automatic process. We also used a diagnostic plot of standardized robust residuals versus robust Mahalanobis distances from the mmregress function (data not shown). Linear regression equations shown in the different scatter plots are those obtained by using the mmregress module of Stata. The heatmap graphics were generated with a script in R [45]. The genes were ordered by the mean value of the log of NLR values. Positive log NLR values are shown in green going from dark green (weak positive) to light green (strong positive) and Negative log NLR values are shown in red going from dark red (weak negative) to light red (strong negative). To compare different experiments, we used modified NLR defined as the ratio between the NLR and the present threshold, the highest value of the NLR mean + SEM among negative controls. Three levels of significance were used for the *P*-value lower than 0.05 (*), 0.01 (**), and 0.001 (***).

## SUPPLEMENTARY MATERIALS FIGURES AND TABLES


