# Supplementary material for: The SRC-family tyrosine kinase HCK shapes the landscape of SKAP2 interactome
Source: Oncotarget. 2018 Feb 6;9(17):13102–15. doi: 10.18632/oncotarget.24424 (PMC5862564; doi:10.18632/oncotarget.24424)
Supplement: Supplementary file 1 [file oncotarget-09-13102-s001.pdf]

The SRC-family tyrosine kinase HCK shapes the landscape of SKAP2 interactome

SUPPLEMENTARY MATERIALS

Supplementary Table 1: NLR of the interactors of SKAP2 labelled at the N-terminal or C-terminal part with (iono) or without (NT) activation by A23187

Interactor	SKAP2N2_NT[a]	SKAP2N2_Iono[a]	SKAP2C2_NT[ab]	SKAP2C2_Iono[ab]	independent upon of the fusion configuration	non validated
APBB1IP	6.57 ± 2.77	21.78 ± 5.79	1.44 ± 0.08	3.11 ± 1.09		X
FAM102A	177.52 ± 25.13	283.94 ± 34.22	12.12 ± 1.11 (7%)	33.95 ± 7.42 (12%)		
FYB	275.57 ± 69.01	1102.80 ± 159.93	299.64 ± 82.56 (108%)	1080.10 ± 279.57 (98%)	X	
NCK1	0.97 ± 0.19	0.90 ± 0.19	0.75 ± 0.21	1.06 ± 0.37		X
NCK2	44.46 ± 11.23	59.86 ± 10.25	3.09 ± 0.79 (7%)	5.78 ± 3.26		
SKAP2	325.71 ± 68.18	609.87 ± 176.84	9.26 ± 1.01 (3%)	21.50 ± 8.37 (4%)		
BLK	40.17 ± 9.47	90.07 ± 13.25	3.18 ± 0.45 (8%)	5.03 ± 1.46		
FGR	5.83 ± 1.59	38.60 ± 7.09	2.12 ± 0.36	2.89 ± 0.54		
FRK	5.57 ± 1.14	27.00 ± 5.01	1.44 ± 0.17	5.35 ± 1.29		
FYN	22.00 ± 4.10	58.86 ± 10.81	2.62 ± 0.61 (12%)	11.60 ± 2.82 (20%)		
HCK	35.25 ± 6.84	49.68 ± 7.56	4.84 ± 0.54 (14%)	13.73 ± 1.99 (28%)		
LCK	46.33 ± 9.71	169.09 ± 28.05	6.51 ±0.76 (14%)	13.55 ± 2.97 (8%)		
LYN	44.07 ± 11.23	103.01 ± 15.68	4.30 ± 0.42 (10%)	14.80 ± 4.65 (14%)		
SRC	18.32 ± 3.50	41.96 ± 8.59	1.91 ± 0.28 (10%)	5.94 ± 1.44		
SRMS	30.09 ± 5.04	71.80 ± 11.49	8.56 ± 0.53 (28%)	9.99 ± 1.81 (14%)		
YES	8.70 ± 1.50	49.10 ± 11.22	2.11 ± 0.14 (24%)	14.43 ± 4.54 (29%)		
FAM102B	4.20 ± 0.80	12.33 ± 1.75	1.46 ± 0.30	6.31 ± 3.30		
PCMT	2.84 ± 0.56	12.15 ± 3.13	1.20 ± 0.08	4.49 ± 1.62		
SGTB	6.34 ± 1.57	19.78 ± 3.85	1.33 ± 0.13	5.02 ± 1.25		
Threshlod	7,95	23,63	1,76	9,61		
[a]mean ± SEM						
[b]percentage of SKAP2N2 value						
Adaptor	SKAP2N2 positive	SKAP2N2 positive	SKAP2C2 positive	SKAP2C2 positive		
SRC family member						
RRS						

Supplementary Table 2: NLR of the interactors of SKAP2 and its DDIM mutant labelled at the N-terminal part with (A) or without (NT) activation by A23187

Interactor	SKAP2N2_NT[a]	DDIMN2_NT[b]	SKAP2N2_A	DDIMN2_A[b]	sensitive to dimerization	less affected by dimerizations
FAM102A	89.70 ± 15.11	53.41 ± 5.22 (60%)	177.02 ± 36.06	81.41 ± 11.17 (46%)		X
FYB	73.89 ± 19.13	47.79 ± 6.72 (65%)	445.06 ± 87.65	172.32 ± 23.59 (39%)		X
SKAP2	132.26 ± 25.24	1.18 ± 0.18	352.65 ± 71.05	1.27 ± 0.19	X	
BLK	16.81 ± 2.63	1.62 ± 0.17	73.51 ± 14.20	4.05 ± 0.57 (6%)		
FGR	5.62 ± 0.98	1.33 ± 0.33	32.76 ± 7.12	2.12 ± 0.26 (6%)		
FRK	6.32 ± 1.13	1.68 ± 0.19	19.46 ± 4.09	1.63 ± 0.14		
FYN	17.83 ± 2.83	2.98 ± 0.53 (17%)	68.48 ± 19.19	3.96 ± 0.36 (6%)		
HCK	20.79 ± 3.13	2.67 ± 0.36	36.27 ± 6.77	4.25 ± 0.47 (12%)		
LCK	26.58 ± 4.06	3.30 ± 0.34 (12%)	73.82 ± 13.11	7.53 ± 0.63		
LYN	28.88 ± 5.43	4.35 ± 0.38 (15%)	74.95 ± 15.55	5.78 ± 0.73 (8%)		
SMRS	27.44 ± 5.40	4.45 ± 0.46 (16%)	42.36 ± 10.63	6.02 ± 0.49 (14%)		
SRC	12.82 ± 2.26	2.38 ± 0.50	28.40 ± 4.74	2.26 ± 0.44 (8%)		
YES	5.22 ± 0.97	1.45 ± 0.25	30.07 ± 6.56	2.97 ± 0.63 (10%)		
FAM102B	7.17 ± 1.01	2.42 ± 0.33	6.48 ± 1.52	1.03 ± 0.08		
ZC3	2.56 ± 0.40	1.04 ± 0.13	9.45 ± 2.11	1.68 ± 0.18		
Threshold	8,18	2,75	11,56	1.86		

[a]mean ± SEM

[b]percentage of SKAP2N2

Adaptor	SKAP2N2 positive	DDIMN2 positive	SKAP2N2_A positive	DDIMN2_A positive
SRC family member				
RRS				

Supplementary Table 3: NLR of the interactor sof SKAP2 and its DDIM mutant labelled at the N-terminal part with (A) or without (NT) activation by A23187

Interactor[a]	SKAP2N2_NT[b]	DDIMN2_NT[c]	SKAP2N2_A	DDIMN2_A[c]	sensitive to dimerization	less affected by dimerization
FAM102A	92.66 ± 11.58	69.80 ± 4.14 (75%)	207.00 ± 37.43	175.13 ± 27.43 (85%)		X
FYB	135.74 ± 12.36	176.82 ± 12.38 (130%)	852.53 ± 79.71	623.14 ± 122.03 (74%)		X
NCK2	22.76 ± 5.89	2.48 ± 0.16 (11%)	64.19 ± 5.84	6.66 ± 1.31 (10%)		
SKAP2	93.36 ± 19.24	1.63 ± 0.13	468.46 ± 83.21	4.35 ± 0.96 (1%)	X	
BLK	73.45 ± 9.63	15.06 ± 1.16 (21%)	118.71 ± 12.45	20.38 ± 3.49 (17%)		
FGR	37.94 ± 7.08	8.76 ± 1.09 (23%)	42.07 ± 6.97	6.71 ± 1.20 (16%)		
FRK	4.81 ± 0.53	1.70 ± 0.29	9.35 ± 1.48	2.36 ± 0.38		
FYN	6.40 ± 0.76	1.76 ± 0.12	30.36 ± 3.20	4.17 ± 0.75 (14%)		
HCK	48.59 ± 11.67	11.75 ± 1.33 (24%)	45.04 ± 5.82	10.16 ± 1.77 (23%)		
LCK	13.10 ± 2.83	3.55 ± 0.39 (28%)	23.57 ± 4.80	5.45 ± 0.99 (23%)		
LYN	49.43 ± 10.07	11.20 ± 1.29 (23%)	46.20 ± 11.53	9.41 ± 2.12 (20%)		
SMRS	8.45 ± 3.52	4.28 ± 0.92 (51%)	30.53 ± 4.43	9.58 ± 1.52 (31%)		
YES	2.14 ± 0.35	1.48 ± 0.13	9.16 ± 1.38	1.89 ± 0.37		
SGTB	1.65 ± 0.43	1.06 ± 0.17	9.81 ± 3.61	2.13 ± 0.39		
FAM102B	5.95 ± 1.11	1.77 ± 0.23	11.69 ± 2.21	2.24 ± 0.38		
Threshold	7.06	2.00	13.90	2.62		

[a]SRC kinases are labelled at the C-Terminal part

[b]mean ± SEM

[c]percentage of SKAP2N2

Adaptor	SKAP2N2 positive	DDIMN2 positive	SKAP2N2_A positive	DDIMN2_A positive
SRC family member				
RRS				

Supplementary Table 4: NLR of the interactors of SKAP2 and its DDIM mutant labelled at the C-terminal part

Interactor	SKAP2C2_NT[a]	DDIMC2_NT[b]	sensitive to dimerization	less affected by dimerization
FAM102A	26.29 ± 4.12	12.19 ± 1.17 (46%)		X
FYB	748.33 ± 178.78	100.80 ± 22.51 (13%)		
NCK1	0.87 ± 0.17	0.73 ± 0.20		
NCK2	2.88 ± 0.53	1.55 ± 0.40		
SKAP2	22.36 ± 6.01	1.28 ± 0.27	X	
BLK	2.95 ± 0.64	1.37 ± 0.24		
FYN	5.58 ± 0.49	1.98 ± 0.39 (35%)		
HCK	7.57 ± 0.46	2.35 ± 0.41 (31%)		
LCK	19.67 ± 2.90	4.79 ± 1.53 (24%)		
LYN	8.30 ± 1.38	2.22 ± 0.44 (27%)		
SMRS	8.21 ± 0.62	1.90 ± 0.12 (23%)		
SRC	2.32 ± 0.16	0.60 ± 0.15		
FAM102B	2.19 ± 0.16	1.21 ± 0.24		
SGTB	2.79 ± 0.20	1.26 ± 0.16		
Threshold	2.99	1.45		

[a]mean ± SEM

[b]percentage of SKAP2C2

Adaptor	SKAP2C2 positive	DDIMC2 positive
SRC family member		
RRC		

Supplementary Table 5: NLR of the interactors of SKAP2 and its DIMPH mutant labelled at the N-terminal part with (A) and without (NT) activation by A23187

Interactor	SKAP2N2_NT[a]	DIMPHN2_NT[b]	SKAP2N2_A	DIMPHN2_A[b]	less affected in the mutant
FYB	741.56 ± 150.10	17.69 ± 0.80 (2%)	735.94 ± 177.24	21.39 ± 2.54 (3%)	X
NCK2	29.91 ± 6.65	4.66 ± 0.38 (16%)	39.55 ± 9.60	4.68 ± 1.08 (12%)	
SKAP2	425.47 ± 87.69	221.58 ± 41.61 (52%)	526.95 ± 96.95	285.61 ± 28.18 (54%)	
BLKN1	67.66 ± 14.26	2.42 ± 0.16 (4%)	89.81 ± 20.85	2.25 ± 0.50 (3%)	X
HCKN1	14.20 ± 2.03	2.59 ± 0.48	27.88 ± 3.96	2.90 ± 0.56 (10%)	
LCKN1	70.88 ± 12.40	2.15 ± 0.17 (3%)	113.63 ± 17.88	1.77 ± 0.47 (2%)	X
LYNN1	75.46 ± 16.38	6.14 ± 1.31 (8%)	105.43 ± 17.32	4.40 ± 0.63 (4%)	X
SMRSN1	54.27 ± 8.07	3.56 ± 0.13 (7%)	67.75 ± 9.72	3.35 ± 0.32 (5%)	X
SRCN1	13.34 ± 3.38	3.35 ± 0.66	23.19 ±4.06	5.20 ± 1.44 (22%)	
BLKC1	108.49 ± 7.97	18.13 ± 0.98 (17%)	77.16 ± 20.22	21.04 ± 5.13 (27%)	
HCKC1	26.08 ± 3.95	14.88 ± 2.61 (57%)	24.69 ± 1.81	16.42 ± 1.20 (66%)	
LCKC1	14.15 ± 75.46	2.07 ± 0.37	21.20 ± 5.08	2.79 ± 0.80 (13%)	
LYNC1	10.87 ± 1.70	4.42 ± 0.65	10.97 ± 0.94	6.78 ±1.10	
SRMSC1	17.27 ± 3.17	2.59 ± 0.65	10.54 ± 3.15	1.84 ± 0.47	
FAM102B	17.10 ± 2.82	2.90 ± 0.23	14.32 ± 2.02	2.50 ± 0.32	
SGTB	7.93 ± 2.65	2.93 ± 0.32	6.54 ± 2.56	2.73 ± 0.40	
Threshold	19.92	3.25	16.34	3.13	

[a]mean ± SEM
[b]percentage of SKAP2N2

Adaptor	SKAP2N2 positive	DIMPHN2 positive	SKAP2N2_A positive	DIMPHN2_A positive	
SRC family memberN1					
SRC family memberC1					
RRS					

Supplementary Table 6: NLR of the interactors of SKAP2 and its DIM mutant labelled at the N-terminal part in competition with either pCNeo or DIMPH and with (A) or without activation by A23197

Interactor[a]	SKAP2N2_pCNeo_NT[b]	DIMN2_DIMPH_NT	SKAP2N2_DIMPH_NT	SKAP2N2_pCNeo_A	DIMN2_DIMPH_A	SKAP2_DIMPH_A
FAM102A	4.42 ± 1.34	16.69 ± 4.59	18.39 ± 4.68	13.42 ± 5.44	21.95 ± 9.61	27.07 ± 10.46
FYB	75.44 ± 9.34	331.91 ± 74.99	198.17 ± 16.66	116.03 ± 19.04	367.98 ± 56.59	200.25 ± 14.19
SKAP2	84.51 ± 4.84	0.98 ± 0.18	117.74 ± 17.00	155.47 ± 19.17	2.64 ± 0.68	188.45 ± 12.68
BLK	34.90 ± 9.91	23.01 ± 7.25	47.45 ± 13.69	22.58 ± 4.24	13.50 ± 2.81	32.46 ± 6.48
FGR	3.67 ± 0.16	2.35 ± 0.47	4.66 ± 0.52	6.95 ± 1.28	3.60 ± 0.65	10.54 ± 1.75
FYN	1.21 ± 0.29	1.25 ± 0.49	2.24 ± 0.52	3.69 ± 1.49	1.53 ± 0.54	5.63 ± 2.05
HCK	21.59 ± 6.98	12.02 ± 2.92	12.08 ± 3.86	15.43 ± 2.17	8.36 ± 0.90	20.30 ± 2.47
LCK	5.99 ± 1.01	4.52 ± 0.56	8.98 ± 0.91	19.96 ± 1.99	12.06 ± 0.83	28.11 ± 1.93
LYN	3.52 ± 0.99	2.11 ± 0.53	5.83 ± 1.83	10.34 ± 2.76	4.25 ± 0.66	12.67 ± 2.22
SRMS	9.11 ± 1.68	12.07 ± 1.76	18.25 ± 3.14	11.39 ± 2.46	8.63 ± 1.65	15.83 ± 3.21
FAM102B	1.58 ± 0.33	1.88 ± 0.18	4.06 ± 0.57	3.66 ± 0.40	3.01 ± 0.59	6.61 ± 0.90
Threshold	1.92	2.06	4.63	4.06	3.6	7.51

[a]SRC kinases are labelled at the C-Terminal part

[b]mean ± SEM

Adaptor	SKAP2N2_pCNeo_NT positive	DIMN2_DIMPH_NT positive	SKAP2N2_DIMPH_NT positive	SKAP2N2_pCNeo_A positive	DIMN2_DIMPH_A positive	SKAP2N2_ DIMPH_A positive
SRC family memberC1						
RRS						

Supplementary Table 7: NLR of the interactors of SKAP2 and its W336F mutant labelled at the C-terminal part with (A) or without (NT) activation by A23187

Interactor[a]	SKAP2C2_NT[b]	W336KC2_NT	SKAP2C2_A	W336KC2_A	sensitive to W336F mutation
FAM102A	15.13 ± 2.79	2.30 ± 0.38	38.24 ± 3.20	4.51 ± 0.43	X
FYB	607.14 ± 132.43	1.49 ± 0.18	2548.43 ± 226.85	3.13 ± 0.38	X
SKAP2	16.35 ± 2.67	11.55 ± 1.16	61.21 ± 2.84	28.22 ± 2.50	
BLK	13.96 ± 2.29	8.91 ± 0.91	16.73 ± 3.30	11.89 ± 1.34	
FGR	4.88 ± 0.63	4.22 ± 0.44	4.38 ± 0.50	2.83 ± 0.30	
FYN	3.30 ± 0.46	4.36 ± 0.45	23.01 ± 4.03	12.81 ± 1.86	
HCK	11.01 ± 0.68	7.05 ± 0.53	18.51 ± 2.61	13.78 ± 1.90	
LCK	5.47 ± 0.96	5.24 ± 0.55	14.72 ± 1.83	9.64 ± 0.74	
LYN	4.87 ± 0.57	3.72 ± 0.41	10.21 ± 1.25	5.87 ± 0.94	
SMRS	5.55 ± 0.57	4.08 ± 0.21	15.30 ± 1.50	10.45 ± 1.04	
YES	1.77 ± 0.51	2.18 ± 0.37	2.42 ± 0.52	1.86 ± 0.26	
FAM102B	4.60 ± 0.62	4.34 ± 0.39	3.83 ± 0.24	2.19 ± 0.21	
PCMT1	1.09 ± 0.17	1.34 ± 0.24	2.96 ± 0.52	1.54 ± 0.18	
Threshold	5.22	4.73	4.07	2.4	

[a]SRC kinases are labelled at the C-Terminal part

[b]mean ± SEM

Adaptor	SKAP2C2_NT Pos.	W336KC2_NT Pos.	SKAP2C2_A Pos.	W336KC2_A Pos.	
SRC family memberC1					
RRS					

Supplementary Table 8: NLR of FAM102A, FYB, NCK2 and their mutants with SKAP2 labelled at the N-terminal part with (A) or without (NT) activation by A23187

interactor	SKAP2N2_NT[ab]	SKAP2N2_A[b]	affected compare to wild-type
FAM102A	163.75 ± 25.45	231.73 ± 41.09	
FAM102AP249L	9.12 ± 0.81 (6%)	15.53 ± 2.69 (7%)	X
FYB	494.71 ± 75.28	687.45 ± 57.21	
FYBDel	5.12 ± 1.03 (1%)	4.91 ± 1.01 (1%)	X
FYBP370A	88.17 ± 20.33 (18%)	89.40 ± 4.79 (13%)	X
NCK2	18.59 ± 2.45	34.89 ± 2.72	
NCK2R311A	12.09 ± 2.19 (65%)	22.47 ± 3.39 (64%)	X
NCK1	4.03 ± 0.68	7.94 ± 0.56	
FAM102B	6.73 ± 1.02	8.59 ± 0.65	
Threshold	7.75	9.24	

[a]mean ± SEM

[b]percentage of wild-type

Adaptor	SKAP2N2_NT positive	SKAP2N2_A positive	

Supplementary Table 9: NLR of the interactors of SKAP2 and its Y75F mutant labelled at tne N-terminal part with (A) and without (NT) activation by A23187

Interactora	SKAP2N2_NT[b]	Y75FN2_NT[c]	SKAP2N2_A	Y75FN2_A[c]	sensitive to Y75F mutation
FAM102A	92.66 ± 11.58	21.26 ± 2.82 (23%)	207.00 ± 37.43	52.01 ± 9.75 (25%)	
FYB	135.74 ± 12.36	25.05 ± 3.68 (18%)	852.53 ± 79.71	135.97 ± 13.65 (16%)	
NCK2	22.77 ± 5.89	1.68 ± 0.34 (7%)	64.18 ± 5.84	6.90 ± 0.71 (11%)	X
SKAP2	93.36 ± 19.24	19.83 ± 2.97 (21%)	468.46 ± 83.21	87.21 ± 9.19 (19%)	
FGR	37.92 ± 6.23	4.78 ± 0.77 (13%)	42.06 ± 6.97	6.98 ± 1.22 (17%)	
FYN	6.40 ± 0.76	1.94 ± 0.30	30.36 ± 3.20	5.94 ± 0.56 (20%)	
HCK	48.59 ± 11.67	8.02 ± 1.11 (17%)	45.04 ± 5.82	9.70 ± 0.92 (22%)	
FAM102B	5.95 ± 1.11	2.45 ± 0.36	11.69 ± 0.56	3.32 ± 0.30	
SGTB	1.04 ± 0.47	0.90 ± 0.15	9.81 ± 3.61	2.82 ± 0.30	
Threshold	7.06	2.81	12.42	3.62	

[a]SRC kinases are labelled at the C-Terminal part

[b]mean ± SEM

[c]percentage of SKAP2N2

Adaptor	SKAP2N2_NT positive	DIMPHN2_NT positive	SKAP2N2_A positive	DIMPHN2_A positive	
SRC family memberC1					
RRS					

Supplementary Table 10: NLR of the interactors of SKAP2 and its 3YF mutant labelled at the C-terminal part with (A) or without (NT) activation by A23187

Interactor[a]	SKAP2C2_NT[b]	3YFC2_NT	SKAP2C2_A	3YFC2_A
FAM102A	6.80 ± 1.19	8.82 ± 1.23 (130%)	22.52 ± 2.77	25.31 ± 2.62 (112%)
FYB	21.17 ± 5.37	19.68 ± 4.72 (93%)	179.35 ± 22.86	135.65 ± 20.34 (76%)
SKAP2	6.96 ± 1.21	9.05 ± 1.50 (130%)	19.11 ± 3.14	13.07 ± 5.67 (68%)
BLK	12.56 ± 2.47	7.57 ± 1.30 (60%)	19.43 ± 5.50	9.97 ± 1.91 (51%)
FGR	1.75 ± 0.77	1.35 ± 0.46	3.92 ± 0.58	3.33 ± 0.37
FRK	1.28 ± 0.32	1.75 ± 0.47	3.20 ± 0.96	1.81 ± 0.43
FYN	1.60 ± 0.31	1.53 ± 0.19	6.76 ± 1.23	6.15 ± 1.03 (91%)
HCK	4.30 ± 1.82	2.95 ± 1.34 (68%)	11.14 ± 2.38	5.44 ± 1.07 (49%)
LCK	5.71 ± 0.93	4.57 ± 0.59 (80%)	5.03 ± 1.26	3.93 ± 0.99 (78%)
LYN	6.31 ± 1.12	3.06 ± 0.39 (48%)	4.74 ± 0.93	3.13 ± 0.53 (66%)
SMRS	4.06 ± 0.47	2.77 ± 0.32 (68%)	10.77 ± 1.75	6.95 ± 0.98 (65%)
YES	1.85 ± 0.73	1.55 ± 0.48	6.42 ± 1.78	3.02 ± 0.37 (47%)
FAM102B	3.53 ± 0.50	4.34 ± 0.52	3.79 ± 0.52	4.45 ± 0.37
SGTB	1.30 ± 0.23	1.14 ± 0.21	1.95 ± 0.61	1.88 ± 0.36
Threshold	4.03	4.86	4.31	4.82

[a]SRC kinases are labelled at the C-Terminal part

[b]mean ± SEM

Adaptor	SKAP2C2_NT Positive	3YC2_NT Positive	SKAP2C2_A Positive	3YC2_A Positive
SRC family memberC1				
RRS				

Supplementary Table 11: NLR of HCK mutants and wild-type interactiong with SKAP2 labelled at the N-terminal part with (A) or without (NT) activation by A23187

Interactor	SKAP2N2_NT[ab]	SKAP2N2_A
HCK	56.72 ± 8.61 (1.0)	67.61 ± 8.60 (1)
HCKP249A	190.23 ± 13.83 (3.3)	169.67 ± 12.69 (2.5)
HCKP249AR171A	102.01 ± 9.72 (1.8)	84.79 ± 8.58 (1.3)
HCKP249AY411F	49.94 ± 5.01 (0.9)	68.86 ± 6.73 (1.0)
HCKP249AY522F	130.68 ± 24.98 (2.3)	116.09 ± 8.83 (1.7)
HCKR171A	359.66 ±48.67 (6.3)	285.89 ± 10.84 (4.2)
HCKW114K	29.66 ± 2.05 (0.5)	25.86 ± 1.60 (0,4)
HCKW114KR171A	91.25 ± 14.44 (1.6)	68.35 ± 4.02 (1.0)
HCKW114FY522F	173.18 ± 24.30 (3.1)	133.07 ± 24.69 (2.0)
HCKY411F	41.89 ± 4.45 (0.7)	32.77 ± 3.80 (0.5)
HCKY411FY522F	123.06 ± 16.16 (2.2)	130.13 ± 7.53 (1.9)
HCKY522F	217.50 ± 15.14 (3.8)	171.30 ± 14.90 (2.5)

[a]mean ± SEM

[b]ratio with HCK

Supplementary Table 12: NLR of the HCK-mutated interactors of SKAP2 and its DDIM mutant labelled at the N-terminal or C-terminal part with (A) or without (NT) activation by A23187

Interactor	SKAP2C2[ab]	SKAP2N2_NT	DDIMN2_NT[c]	SKAP2N2_A	DDIMN2_A[c]
FAM102A		92.26 ± 7.86	102.85 ± 23.35 (111%)	131.86 ±10.67	155.85 ± 18.16 (118%)
FYB	508.06 ± 97.14	397.36 ± 116.85	557.44 ± 208.95 (140%)	745.89 ± 70.05	1504.98 ± 229.38 (202%)
SKAP2	22.67 ± 4.77	238.73 ± 35.67	2.29 ± 0.99 (1%)	325.26 ± 37.56	4.92 ± 1.68 (2%)
HCK	3.17 ± 0.57 (1.0)	14.32 ± 4.44	7.22 ± 2.80 (50%)	31.65 ± 3.38	15.90 ± 2.78 (50%)
HCK-P249A	18.15 ± 2.89 (5.7)	62.34 ± 6.87	40.33 ± 14.04 (65%)	145.69 ± 17.41	97.40 ± 10.50 (67%)
HCK-P249AR171A	25.65 ± 2.02 (8.1)	81.53 ± 11.63	49.15 ± 16.37 (60%)	84.04 ± 7.98	93.22 ±11.72 (111%)
HCK-P249AY411F	5.29 ± 0.47 (1.7)	19.86 ± 3.84	15.83 ± 6.45 (80%)	32.09 ± 4.47	33.23 ± 5.42 (103%)
HCK-P249AY522F	23.94 ± 2.76 (7.6)	84.17 ± 10.07	53.36 ± 17.70 (63%)	102.48 ± 11.47	108.82 ± 12.19 (106%)
HCK-R171A	30.17 ± 2.04 (9.5)	139.30 ± 16.92	95.27 ± 32.73 (68%)	170.97 ± 12.60	204.83 ± 28.39 (120%)
HCK-W114K	2.71 ± 0.48 (0.9)	9.12 ± 1.89	5.21 ± 2.15 (57%)	13.32 ± 1.53	8.35 ± 1.11 (63%)
HCK-W114KY522F	57.03 ± 2.69 (18.0)	143.13 ± 17.48	76.00 ± 22.99 (53%)	176.93 ± 11.97	127.94 ± 15.84 (72%)
HCK-Y411F	5.02 ± 0.42 (1.6)	10.23 ± 1.36	6.75 ± 2.68 (66%)	10.68 ± 1.64	9.69 ± 1.24 (91%)
HCK-Y522F	26.69 ± 2.35 (8.4)	76.68 ±9.87	47.83 ± 15.61 (62%)	96.87 ± 6.55	89.10 ± 12.51 (92%)
HCK-Y411FY522F	22.52 ± 1.94 (7.1)				
FAM102B	2.25 ± 0.36	4.99 ± 0.92	1.64 ± 0.58	5.19 ± 0.28	2.34 ± 0.24
SGTB	1.72 ± 0.49	3.00 ± 0.70	1.56 ± 0.42	3.60 ± 0.61	1.55 ± 0.37
Threshold	2.61	5.91	2.22	5.47	2.58

[a]mean ± SEM

[b]percentage of HCK

[c]percentage of SKAP2N2

Adaptor	SKAP2C2 Pos.	SKAP2N2_NT Pos.	DDIMN2_NT Pos.	SKAP2N2_A Pos.	DDIMN2_A Pos.
SRC family member					
RRS					

Supplementary Table 13: Sequences of primers

SKAP2_without_TGA_Forward	GGGGACAACTTTGTACAAAAAAGTTGGCAT
SKAP2_without_TGA_Reverse	GGGGACAACTTTGTACAAGAAAGTTGGGTAAATATCATACATCTCCATTATGTA
SKAP2_DIMPH_Reverse	GGGGACAACTTTGTACAAGAAAGTTGGGTACTACTATTCCATATCTTGCAATACAAATT
SKAP2_DIM_Forward[a]	GGAACCTGTTGGCAGATGaTGAAACAgaTGaAGCAGATATACTGAAAGG
SKAP2_DIM_Reverse	CCTTTCAGTATATCTGCTtCAtcTGTTTCAtCATCTGCCAACAGGTTCC
SKAP2_W336K_Forward	GCAAGGAATACAATAGATATGGCaaGTGGGTAGGAGAAATGAAGG
SKAP2_W336K_Reverse	CCTTCATTTCTCCTACCCACttGCCATATCTATTGTATTCCTTGC
SKAP2_Y238F_Forward	GAGAGAGGAGAATTATtTGATGATGTTGATCATCCTCTACC
SKAP2_Y238F_Reverse	GGTAGAGGATGATCAACATCATCAaATAATTCTCCTCTCTC
SKAP2_Y261F_Forward	GCAGTCAACCAATAGATGATGAAATTTtTGAAGAACTTCCAGAAGAAGAAGAGG
SKAP2_Y261F_Reverse	CCTCTTCTTCTTCTGGAAGTTCTTCAaAAATTTCATCATCTATTGGTTGACTGC
SKAP2_Y75F_Forward	GCAGAAGATGGGGAAGAATtTGATGACCCTTTTGC
SKAP2_Y75F_Reverse	GCAAAAGGGTCATCAaATTCTTCCCCATCTTCTGC
FAM102A-P149L-Forward	CCCGGAGAAGCtGCCGCGGCCAC
FAM102A-P149L-Reverse	GTGGCCGCGGCaGCTTCTCCGGG
FYB_Del_Forward	GAATTCAGCCACCCCGAATGTTGACCTGACG
FYB_Del_Reverse	CGTCAGGTCAACATTCGGGGTGGCTGAATTC
FYB_P370A_Forward	CACCTCCACCAAAAgCCAACAGACCACCAAATG
FYB_P370A_Reverse	CATTTGGTGGTCTGTTGGcTTTTGGTGGAGGTG
HCK_P249A_Forward	CCAAGCCCCAGAAGgCTTGGGAGAAAGATG
HCK_P249A_Reverse	CATCTTTCTCCCAAGcCTTCTGGGGCTTGG
HCK_R171A_Forward	GGTCTCGCTATCCgcGATCATGAAGGAGCCC
HCK_R171A_Reverse	GGGCTCCTTCATGATCgcGGATAGCGAGACC
HCK_W114K_Forward	GGAATCCGGGGAGaaGTGGAAGGCTCGATCC
HCK_W114K_Reverse	GGATCGAGCCTTCCACttCTCCCCGGATTCC
HCK_Y411F_Forward	CATTGAGGACAACGAGTtCACGGCTCGGGAAG
HCK_Y411F_Reverse	CTTCCCGAGCCGTGaACTCGTTGTCCTCAATG
HCK_Y522F_Forward	CCACAGAGAGCCAGTtCCAACAGCAGCC
HCK_Y522F_Reverse	GGCTGCTGTTGGaACTGGCTCTCTGTGG
NCK2_R311A_Forward	GGCGACTTCCTCATTgcGGACAGCGAGTCC
NCK2_R311A_Reverse	GGACTCGCTGTCCgcAATGAGGAAGTCGCC

[a]The mutated base is lower case.

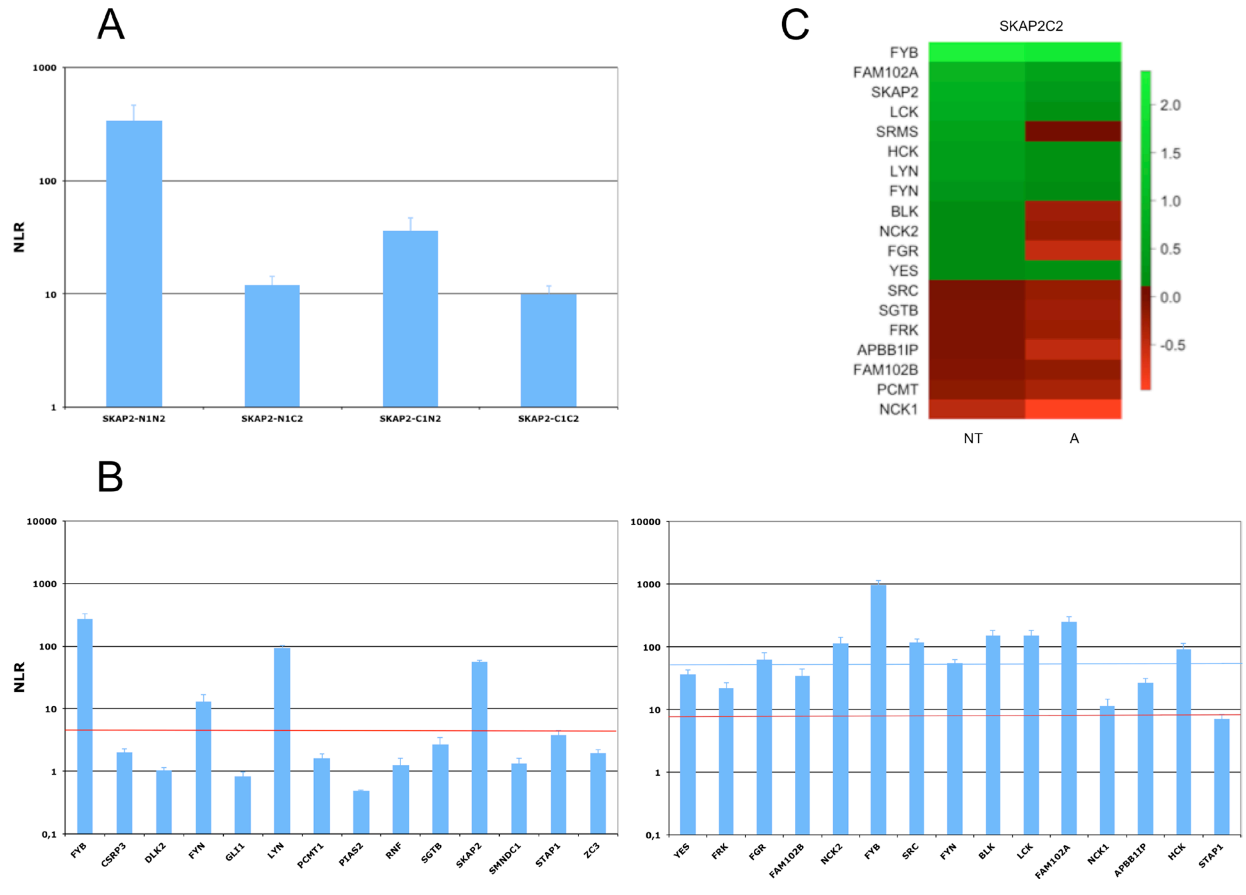

Supplementary Figure 1: Development of the luciferase complementation assay. (**A**) Normalized luminescence ratio of the four SKAP2 pairs. SKAP2 protein is fused at the N-terminal or the C-terminal with either hemi-luciferase 1 or 2. (**B**) Accuracy and sensitivity of the PPI detection. They were evaluated first using a positive or interacting set of four proteins, FYB, FYN, NCK2, SKAP2 and an a priori non-interacting set of 10 random human proteins (CRSP3, DLK2, GLI1, PCMT1, PIAS2, RNF, SGTB, SMNDC1, STAP1, ZC3) (left panel) and secondary using the same interacting set, the 11 putative interactors (YES, FRK, FGR, FAM102B, SRC, BLK, LCK, FAM102A, NCK1, APBB1IP, HCK) and protein STAP1, which has the highest NLR among the set of a priori non-interacting proteins. All proteins are labelled at the N-terminal with hemi-luciferase 1 or 2. Threshold for a priori negative controls: red line. Threshold for positive controls: blue line. (**C**) Heatmap showing the protein-protein interactions of SKAP2C2 with putative partners detected by luciferase complementation assay. Scoring is based on log-transformation of normalized relative luminescence (NLR) intensity and the null value corresponds to the threshold. Interacting pairs are ranked high to low with strongest in green and lowest in red. Interactions are monitored on non-stimulated (NT) or A23187 calcium ionophore activated (A) cells.

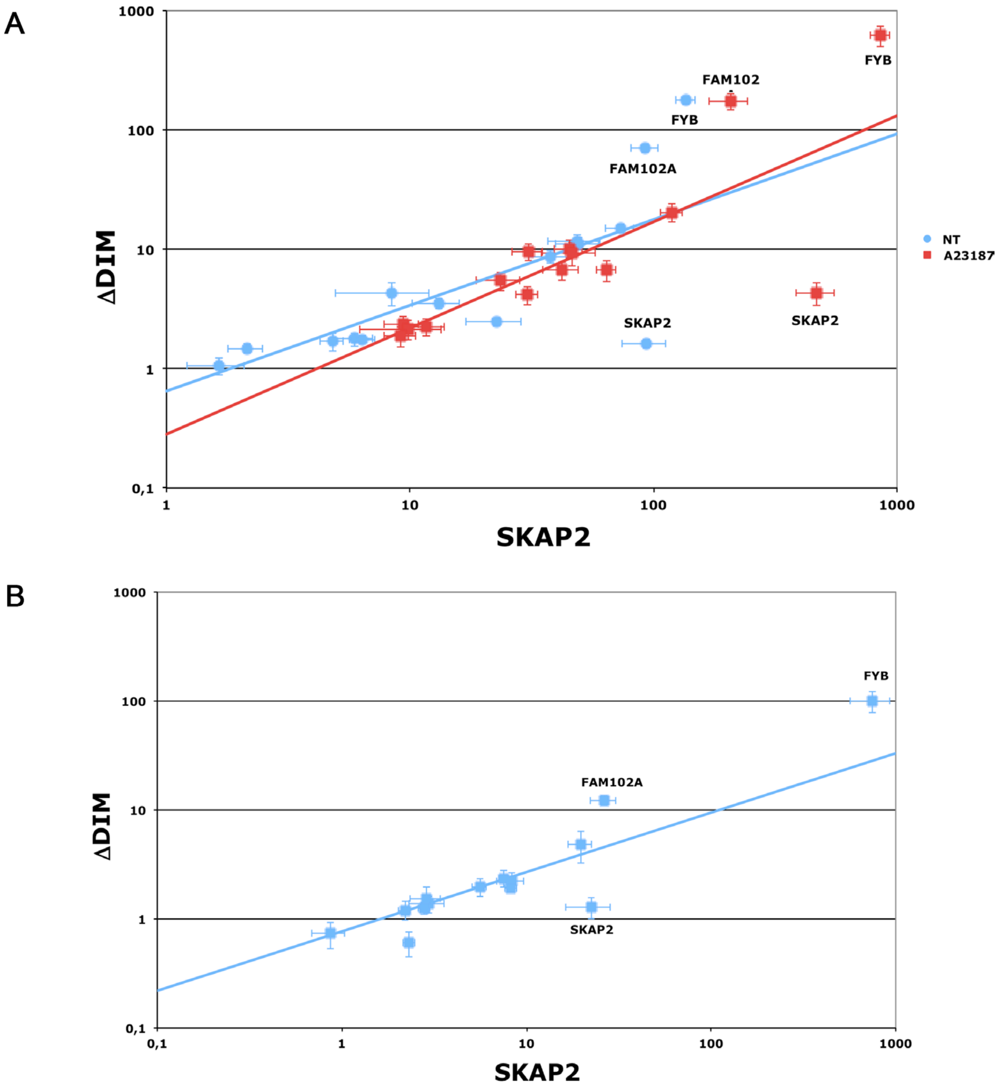

Supplementary Figure 2: Localization of the fused hemi luciferase protein does not affect the role of SKAP2 dimerization on its interactome. Legends are similar to those of Figure 3. (**A**) Scatterplot comparing the interactome of SKAP2N2 and its N2-fused ΔDIM mutant with C1-fused SRC family members. Linear regression equations are respectively $\log_{10}(\Delta DIM) = 0.720\ {}^{*}\log_{10}(SKAP2)-0.187$ for samples without A23187 stimulation (blue line) and $\log_{10}(\Delta DIM) = 0.891\ {}^{*}\log_{10}(SKAP2) - 0.549$ for samples with A23187 stimulation (red line). (**B**) Scatterplot comparing the interactome of SKAP2C2 and its C2-fused ΔDIM mutant. Linear regression equation is $\log_{10}(\Delta DIM) = 0.546\ {}^{*}\log_{10}(SKAP2) - 0.113$.

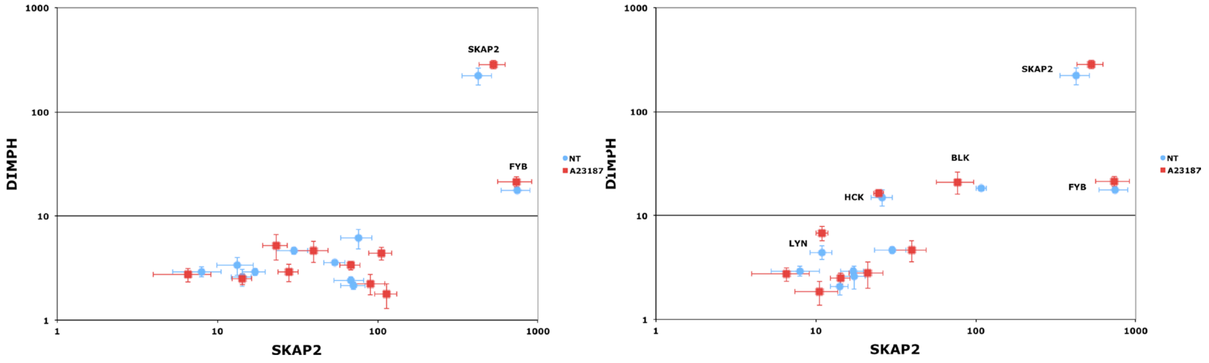

Supplementary Figure 3: Characterization of the DIMPH SKAP2 mutant. Scatterplots compare the interactome of SKAP2 and its DIMPH mutant. SRC family members are fused with the hemi- luciferase either on N-terminal (left panel) or on C-terminal (right panel). Figure of the left panel has been previously shown as Figure 3B.

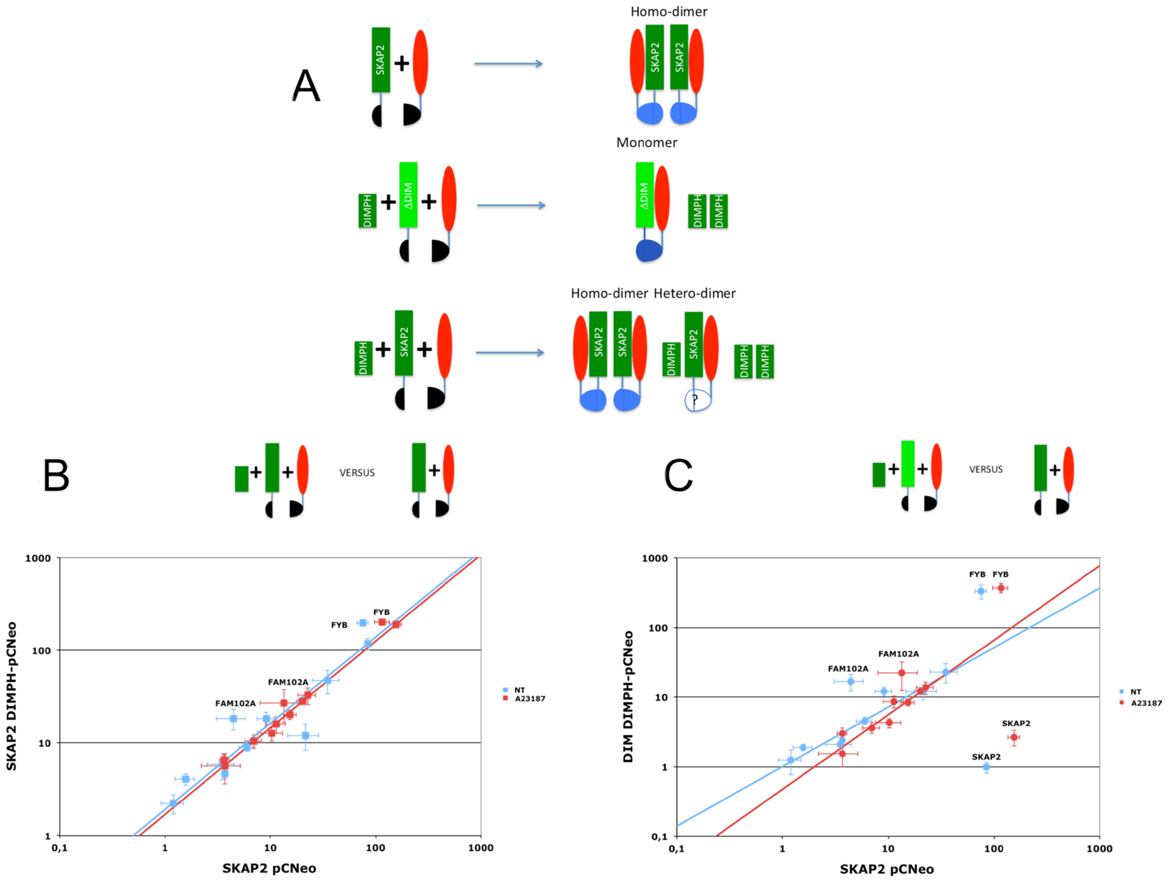

Supplementary Figure 4: (**A**) PPI competition assay principle. Three transfection experiments are done in parallel: The first one associating SKAP2 (dark green large rectangle) hemi-luciferase fusion protein (dark hemi-circle) with its putative hemi-luciferase fused partners (red ellipsoid); The second one with hemi-luciferase fused ΔDIM mutant (light green large rectangle), its hemi-luciferase fused putative partners and DIMPH mutant (dark green small rectangle); The last one with hemi-luciferase fused SKAP2, its hemi-luciferase fused putative partners and DIMPH mutant. We previously showed that PPIs among partners are different depending on SKAP2 (light blue color for luciferase) and its ΔDIM mutant (dark blue color for luciferase) and we would know if it is also the case for hetero-dimer compare to homo-dimer. The hemi-luciferase 2 is fused on N-terminal as in Figure 3A. (**B**) Scatterplot comparing SKAP2 transfected with DIMPH pCNeo vector (y-axis) and SKAP2 transfected with pCNeo vector (x-axis). Linear regression equations are respectively $\log_{10}$(SKAP2 DIMPHpCNeo) = 0.929 $^*\log_{10}$(SKAP2 pCNeo) + 0.281 for samples without A23187 stimulation (blue line) and $\log_{10}$(SKAP2 DIMPHpCNeo) = 0.935 $^*\log_{10}$(SKAP2 pCNeo) + 0.225 for samples with A23187 stimulation (red line). (**C**) Scatterplot comparing ΔDIM SKAP2 mutant transfected with DIMPH pCNeo vector (y-axis) and SKAP2 transfected with pCNeo vector (x-axis). As mentioned previously, data from Supplementary Figure 4B and 4C are from the same experiment. Linear regression equations are respectively $\log_{10}$(ΔDIM DIMPHpCNeo) = 0.855 $^*\log_{10}$(SKAP2 pCNeo) + 0.003 for samples without A23187 stimulation (blue line) and $\log_{10}$(ΔDIM DIMPHpCNeo) = 1.073 *log10(SKAP2 pCNeo)-0.329 for samples with A23187 stimulation (red line).

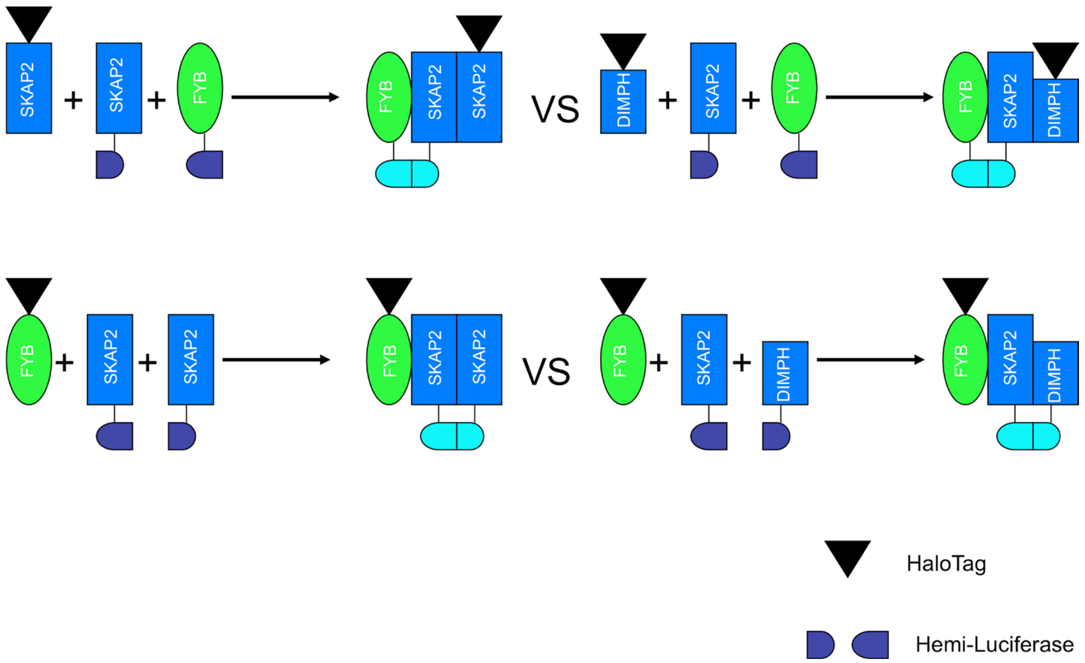

Supplementary Figure 5: Detection of ternary complex principle by using Halotag technology. Either SKAP2 or its DIMPH mutant or FYB is fused to HaloTag as a standardized "hook", used to capture on HaloLink™ resin in which a HaloTag ligand has been covalently bound. Co-expression with the two other proteins fused respectively with each of the two forms of hemi-luciferase generates a luciferase signal. The binding of FYB to the homodimer SKAP2 and the heterodimer DIMPH-SKAP2 is compared using their respective luciferase signal.

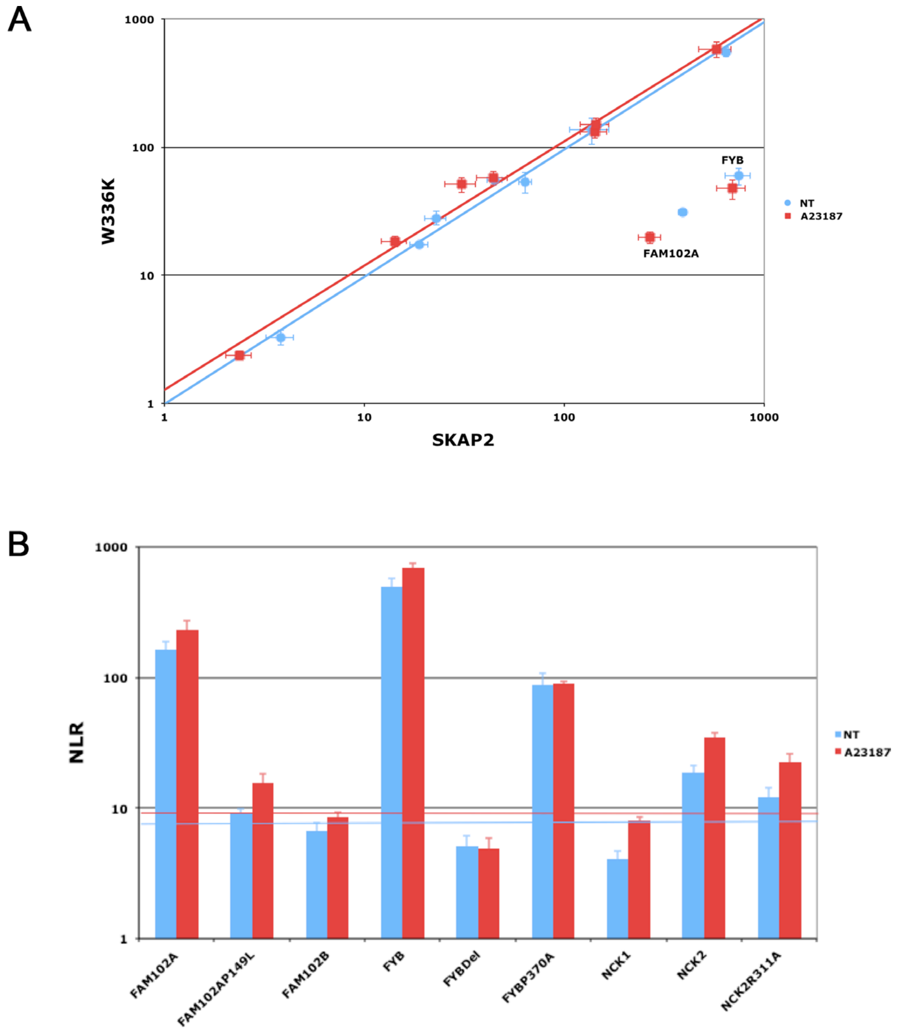

Supplementary Figure 6: Validation of the domains and binding motifs used in the interaction with SKAP2 protein. (**A**) Scatterplot comparing the interactome of SKAP2N1 and its N1-fusedW336K mutant. To increase the sensitivity of the assay, N1-fused HCK mutants replaced N1-fused SRC family members. Legends are similar to those of Figure 3. Linear regression equations are respectively $\log_{10}$(W336K) = 0.992 $^{*}\log_{10}$(SKAP2) −0.003 for samples without A23187 stimulation (blue line) and $\log_{10}$(W336K) = 0.970 $^{*}\log_{10}$(SKAP2) + 0.104 for samples with A23187 stimulation (red line). (**B**) 2D column graph comparing normalized luminescence ratio (NLR) of FYB, FAM102A, and NCK2 with those of their mutants. The hemi-luciferase 1 and 2 are fused on N-terminal. FAM102B is used as negative control. Thresholds for detecting positive PPI are shown for non-stimulated (blue line) or stimulated with A23187 calcium channel (red line) cells.

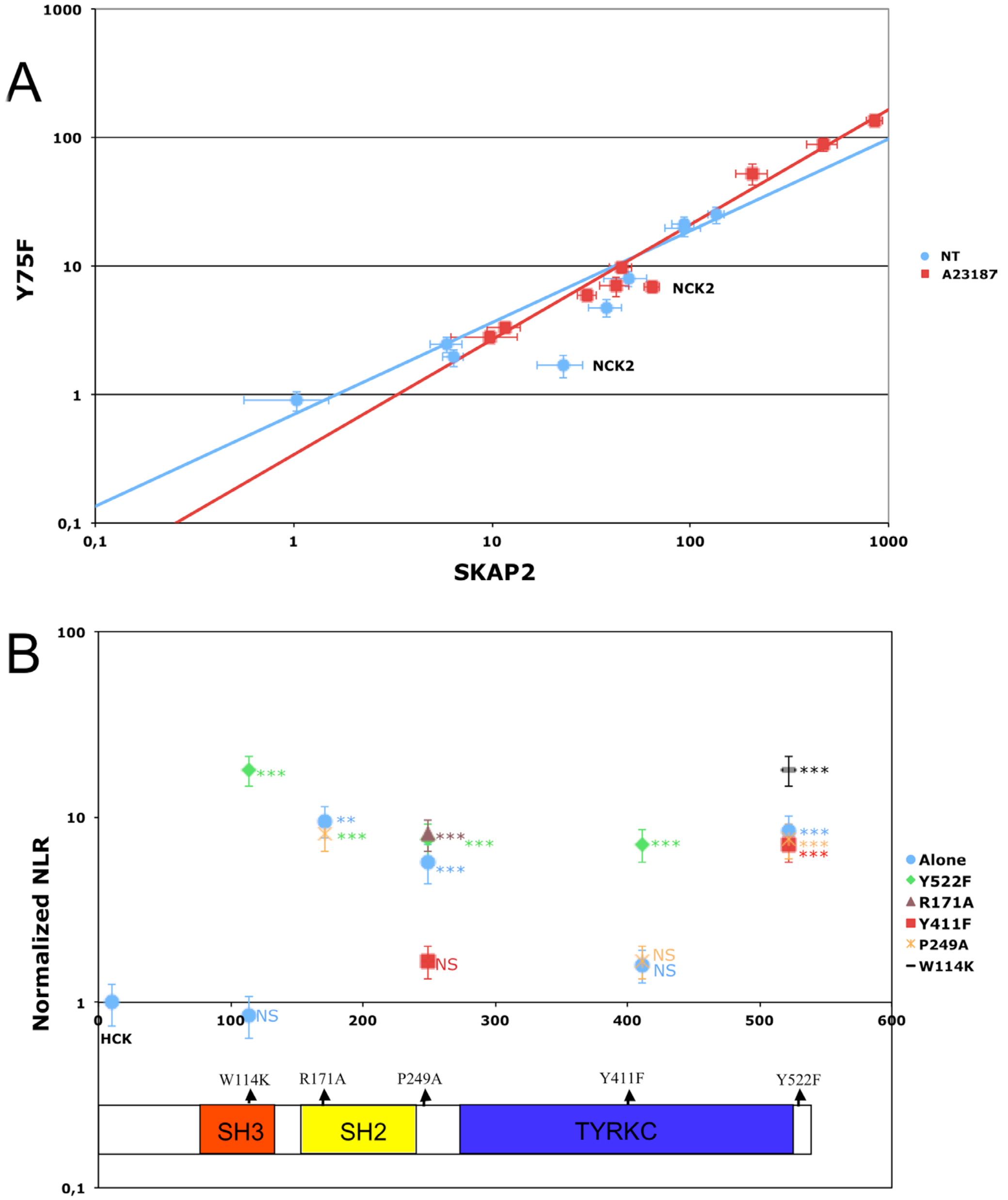

Supplementary Figure 7: (**A**) Role of Tyrosine 75 of SKAP2 on the binding of NCK2 adaptor. Scatterplot compare the interactome of SKAP2N2 and its N2-fused Y75F mutant. Legends are similar to those of Figure 3. Linear regression equations are respectively $\log_{10}$(Y75F) = 0.716 $^{*}\log_{10}$(SKAP2) −0.154 for samples without A23187 stimulation (blue line) and $\log_{10}$(Y75F) = 0.894 $^{*}\log_{10}$(SKAP2) −0.467 for samples with A23187 stimulation (red line). (**B**) Localization of the fused hemi luciferase protein does not affect the role of HCK domain and binding motif inactivations on SKAP2 interactome. Legends are similar to those of Figure 5A. The PPI-mutation plot shows NLR of each HCK mutant according to the position of the mutation without A23187 molecules. SKAP2 protein is fused on the C-terminal with hemi-luciferase 2.

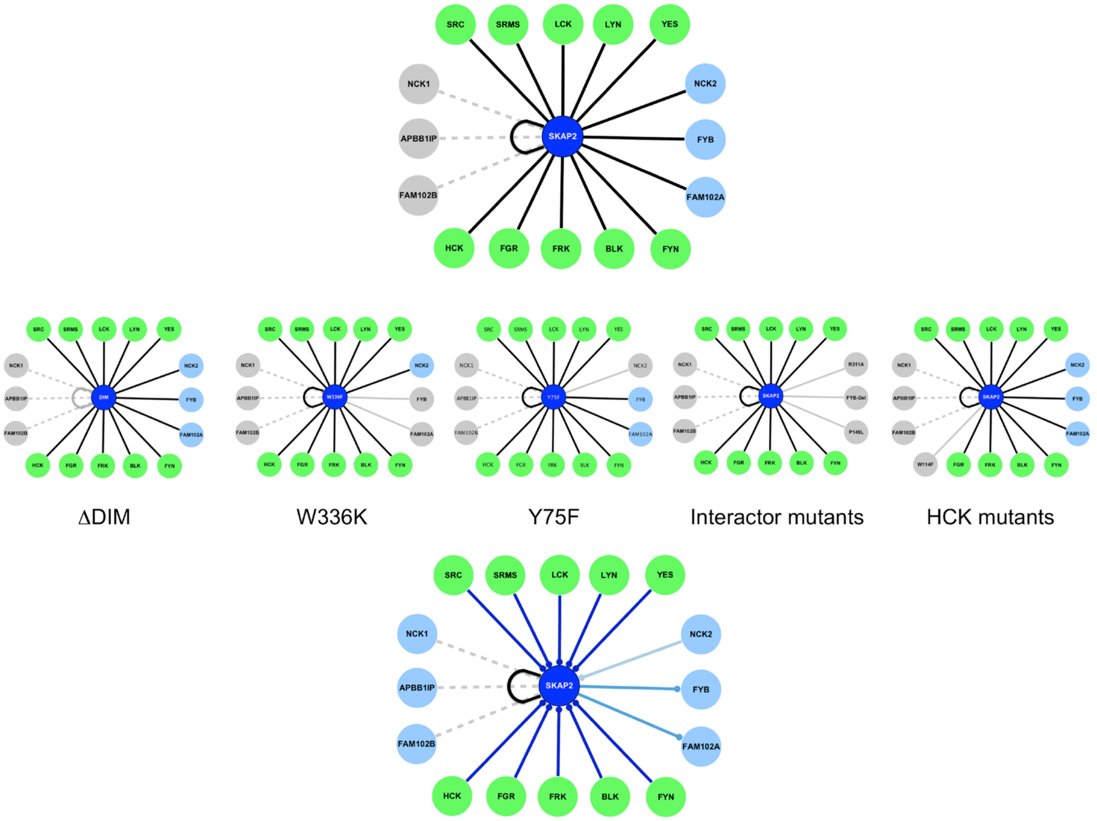

Supplementary Figure 8: Cytoscape schematic of SKAP2 protein-protein interactions (PPI) interactome network highlighting domains and their binding motifs. Node color indicates SRC family members (green), adaptors (blue) and non-interacting proteins (grey). Upper panel: PPIs that have been recovered are indicated with black edge, and lack of interaction with grey dotted edge. Middle panels: PPIs of the different SKAP2 and interactor mutants. Lower panel: Summary of the different domains and binding motifs affecting these PPIs. Target arrow circle indicates node bearing the binding motif. Edge color indicates the nature of the domain interaction. Light blue: interaction with a SH2 domain; Blue: interaction mainly using a SH3 domain; Dark blue: interaction partially using a SH3 domain.
